# Dynamics of bulk and surface oxide evolution in copper foams for electrochemical CO_2_ reduction

**DOI:** 10.1038/s42004-024-01151-0

**Published:** 2024-03-28

**Authors:** Fan Yang, Shan Jiang, Si Liu, Paul Beyer, Stefan Mebs, Michael Haumann, Christina Roth, Holger Dau

**Affiliations:** 1 Department of Physics, Freie Universität Berlin, Arnimallee 14, Berlin, 14195 Germany; 2 Electrochemical Process Engineering, Universität Bayreuth, Universitätsstraße 30, Bayreuth, 95447 Germany

**Keywords:** Electrocatalysis, Electrocatalysis

## Abstract

Oxide-derived copper (OD-Cu) materials exhibit extraordinary catalytic activities in the electrochemical carbon dioxide reduction reaction (CO_2_RR), which likely relates to non-metallic material constituents formed in transitions between the oxidized and the reduced material. In time-resolved operando experiment, we track the structural dynamics of copper oxide reduction and its re-formation separately in the bulk of the catalyst material and at its surface using X-ray absorption spectroscopy and surface-enhanced Raman spectroscopy. Surface-species transformations progress within seconds whereas the subsurface (bulk) processes unfold within minutes. Evidence is presented that electroreduction of OD-Cu foams results in kinetic trapping of subsurface (bulk) oxide species, especially for cycling between strongly oxidizing and reducing potentials. Specific reduction-oxidation protocols may optimize formation of bulk-oxide species and thereby catalytic properties. Together with the Raman-detected surface-adsorbed *OH and C-containing species, the oxide species could collectively facilitate *CO adsorption, resulting an enhanced selectivity towards valuable C_2+_ products during CO_2_RR.

## Abstract (web summary)

Oxide-derived copper materials display high catalytic activities for the electrochemical reduction of carbon dioxide, but the mechanisms surrounding this high performance are not fully understood. Here, the authors use time-resolved operando spectroscopy to probe the structural dynamics of copper oxide reduction and reformation, both in the bulk and on the surface of copper foam catalysts.

## Introduction

The increasing level of atmospheric carbon dioxide (CO_2_) and limited fossil fuel reserves are motivating intensive studies on electrochemical conversion of CO_2_ into value-added chemicals and fuels^1–5^. Particularly, multi-carbon (C_2+_) products are attractive due to their high energy densities and economic values^6–8^. Copper-based materials have attracted significant attention as catalysts for the electrochemical CO_2_ reduction reaction (CO_2_RR), with oxide-derived copper (OD-Cu) catalysts being particularly interesting because of their superior selectivity for C_2+_ product formations when compared to metallic Cu catalysts^9–11^.

Previous studies have suggested that the presence of oxygen species in OD-Cu catalysts plays a particular role in enhancing the activity of CO_2_RR towards C_2+_ products^12–15^. This observation is supported by density functional theory modeling and experimental spectroscopy data^16–18^, which demonstrate that the asymmetry in CO adsorption energies between metallic and oxidized copper sites promotes CO dimerization and is crucial for C_2+_ products formation. However, in this context the existence and origin of copper oxides at catalytic potentials is still debated. For example, a so-called multihollow Cu_2_O catalyst has been shown to exhibit high C_2+_ products generation during CO_2_RR. This high activity was partially attributed to the presence of retained Cu^+^ species, which were detected using operando Raman and X-ray absorption spectroscopy (XAS) approaches^12^. In the case of plasma-activated Cu catalysts, XAS data revealed the practically exclusive presence of metallic Cu after extended CO_2_RR, whereas electron microscopy still found the presence of an oxygen-rich layer^13^. Similarly, the presence of residual oxides was not detected in the OD-Cu catalysts using in-situ XAS^19^ (Here and in the following we do not discriminate between ‘in-situ’ and ‘operando’). The authors noted that XAS is not a surface-sensitive technique; thus, a residual oxide layer in the order of a few nanometers would not have been detected. Interestingly, in their following study^20^, in-situ ambient pressure X-ray photoelectron spectroscopy (APXPS) and quasi-in-situ electron energy loss spectroscopy together revealed the existence of subsurface oxygen. Here, we intend to address these debated issues by a combination of bulk-sensitive and surface-sensitive operando techniques, namely XAS^13,21^ and surface-enhanced Raman spectroscopy (SERS)^22–24^, to differentiate between the dynamics of bulk and surface oxide species in OD-Cu catalysts.

Pulsed electrolysis can effectively enhance the formation of C_2+_ products compared to static CO_2_RR conditions, also leading to increased stability and durability of the catalytic operation^25–31^. In such studies a rectangular potential pulse sequence was used, in which the cathode is held at a reducing potential for a certain time and is then stepped to an oxidizing potential for another time period. It has been suggested that the pulsing approach can be used to control the reaction environment, such as reconstruction of electrode surfaces, formation of surface and subsurface oxides, removal of poisoning species, adsorption of intermediates and control of the interfacial pH^30,32–34^. This motivates our investigation on the chemical nature of oxide species in Cu catalysts and their response to changes in potential, which are assessed in the time domain from seconds to tens of minutes by operando time-resolved XAS and Raman spectroscopy.

In our study we track the oxide evolution of OD-Cu catalysts in real-time under CO_2_RR conditions with operando XAS and Raman, disclosing the bulk and surface composition of OD-Cu catalysts at different potentials, showing that oxides evolution on the surface is significantly faster than in the bulk of the material, and providing evidence for kinetic trapping of copper oxides under negative potentials. Through time-resolved detection of X-ray signals during (i) potential jumps and (ii) cyclic voltammetry (CV), we assess the oxide formation/accumulation dynamics of the bulk material. By employing isotope and buffer exchanges in Raman experiments, we identify the surface species in the Cu oxides/hydroxides spectral region as adsorbed *OH and C-containing intermediates. Our findings contribute to the understanding of the dynamics of the chemical state of OD-Cu catalysts in both the surface and bulk regions during CO_2_RR.

## Results and discussion

Cu electrodes were prepared using the dynamic hydrogen bubble template method as described in the Methods Section. Their porous structure (Supplementary Fig. 1) and properties were similar to that reported in our previous studies^22,23,35^; the material is further on denoted as Cu foam. The overall CO_2_RR performance of the Cu foam is shown in Supplementary Fig. 2. We note that the used Cu foams show enhanced C_2+_ selectivity for a range of applied potentials (e.g., 36% vs. 7% at −0.9 V) when compared to blank Cu foil^35^. All electric potentials in this study refer to the reversible hydrogen electrode (RHE) scale, i.e., −0.9 V = −0.9 V_RHE_.

Powder X-ray diffraction (XRD) was utilized to examine the crystalline structure of the as-prepared and after-electrolysis Cu foams (Supplementary Fig. 3). The as-prepared Cu foam exhibited mainly the characteristic reflections for the Cu_2_O and metallic Cu, with a small contribution from CuO. After electrolysis, metallic Cu became the dominant phase in the foam. To obtain further structural information, especially for XRD-invisible, non-crystalline Cu fractions, the oxide content and oxide evolution in these Cu foams were investigated using operando spectroscopic techniques, as described in the following.

### Operando XAS characterization

#### Types of Cu foam samples

Operando X-ray absorption near-edge structure (XANES) and extended X-ray absorption fine structure (EXAFS) spectroscopy were used to explore redox state and atomic-level structure of the Cu foams. Three types of Cu foams with variable Cu/Cu^+^/Cu^2+^ ratios were prepared for operando XAS experiments, namely by i) electrodeposition on graphene sheets at 1 A cm^−2^ for 10 s, 20 s, or 30 s followed by drying in air for 24 h, ii) electrodeposition as for i) followed by heat-treatment at 200 °C for 5 h, and iii) electrodeposition as for i) followed by storage in KHCO_3_ for one week to obtain copper carbonate hydroxides (CuCarHyd).

#### Oxide classification

**Fig. 1:**
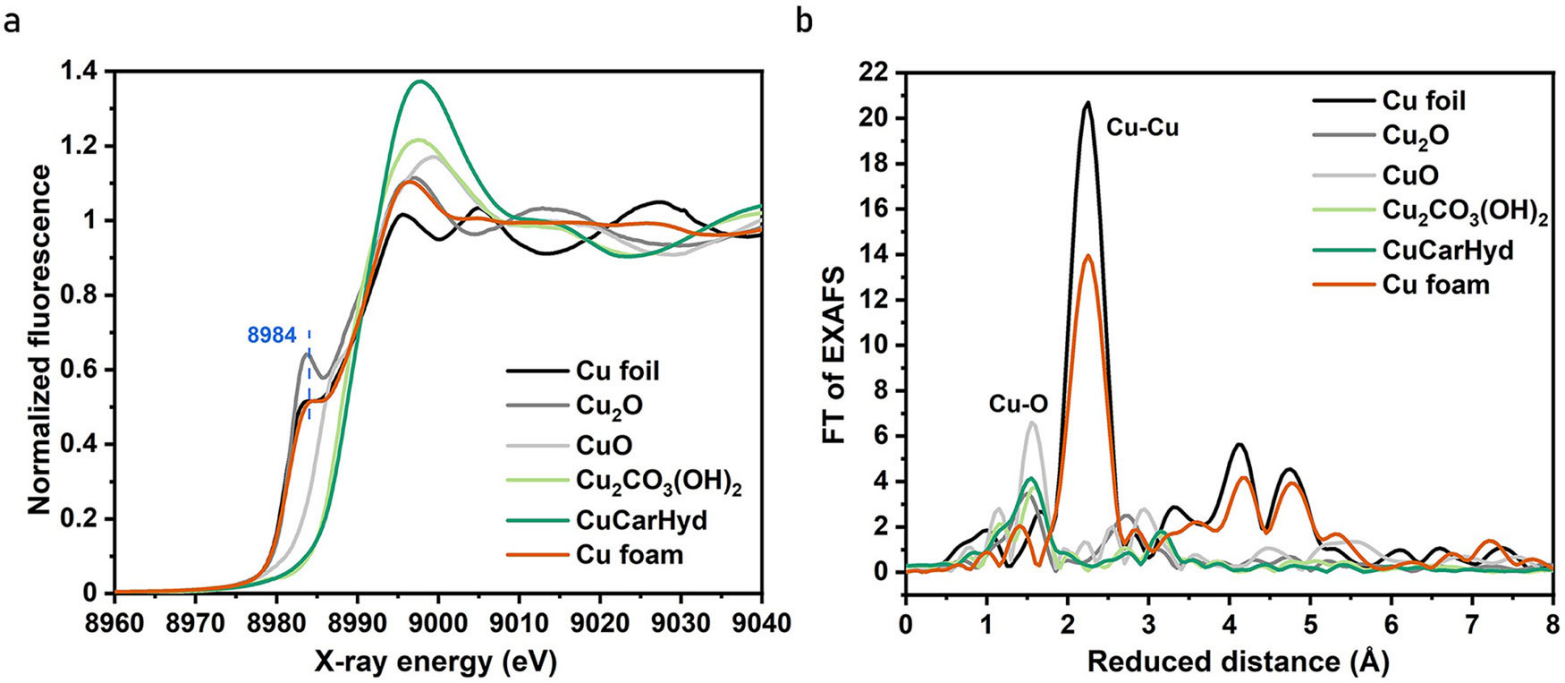
XAS characterization of as-prepared Cu catalysts. The Cu foam was deposited on graphene sheet at 1 A cm^-2^ for 20 s. **a** Cu K-edge XANES and **b** EXAFS spectra of as-prepared Cu foam (orange line), copper carbonate hydroxides (CuCarHyd, green line), as well as of Cu foil (black line), Cu_2_O (gray line), CuO (light gray line), and Cu_2_CO_3_(OH)_2_ (light green line) as references. Pre-edge features at 8984 eV are marked by a dashed line in (**a**).

Figure 1a shows the Cu K-edge XANES spectra of as-prepared Cu foam and CuCarHyd at open circuit potential (OCP), as well as spectra of reference compounds. The XANES spectrum of the Cu foam qualitatively resembles the Cu-foil, but a larger K-edge maximum and smaller post-edge amplitudes in combination with a still similar amplitude of the edge feature around 8984 eV suggest the presence of significant amounts of oxidic species and in particular Cu_2_O within the foam. The XANES spectrum of CuCarHyd is similar to the Cu_2_CO_3_(OH)_2_ reference, suggesting the formation of copper-carbonate. The EXAFS spectra shown in Fig. 1b and Supplementary Fig. 4 support our attributions of chemical species in the Cu foam and CuCarHyd samples. A linear combination of the Cu_2_O and the reduced Cu foam (denoted as Cu^0^) spectra could reasonably account for the XANES data of the Cu foams, including the heat-treated samples (Supplementary Fig. 5 and Supplementary Table 1; the reduced Cu foam was used as a reference here, assuming a fully metallic character but a lower degree of crystallinity compared to the Cu foil; EXAFS flattening effects, however, might not be fully ruled out). Extending the deposition time diminishes the relative Cu_2_O content by up to 20%, likely because air oxidation within the bulk of the thicker foams is retarded, whereas heat-treatment increases the Cu_2_O contents compared to the as-prepared Cu foams by 15-25%. The XANES of CuCarHyd (which likely is not a foam structure any longer) can be well modeled using close to 100% Cu_2_CO_3_(OH)_2_, suggesting near-quantitative transformation into Cu_2_CO_3_(OH)_2_ for this sample type (Supplementary Fig. 6).

#### Reduction of copper oxides at moderately negative potential (−0.2 V)

**Fig. 2:**
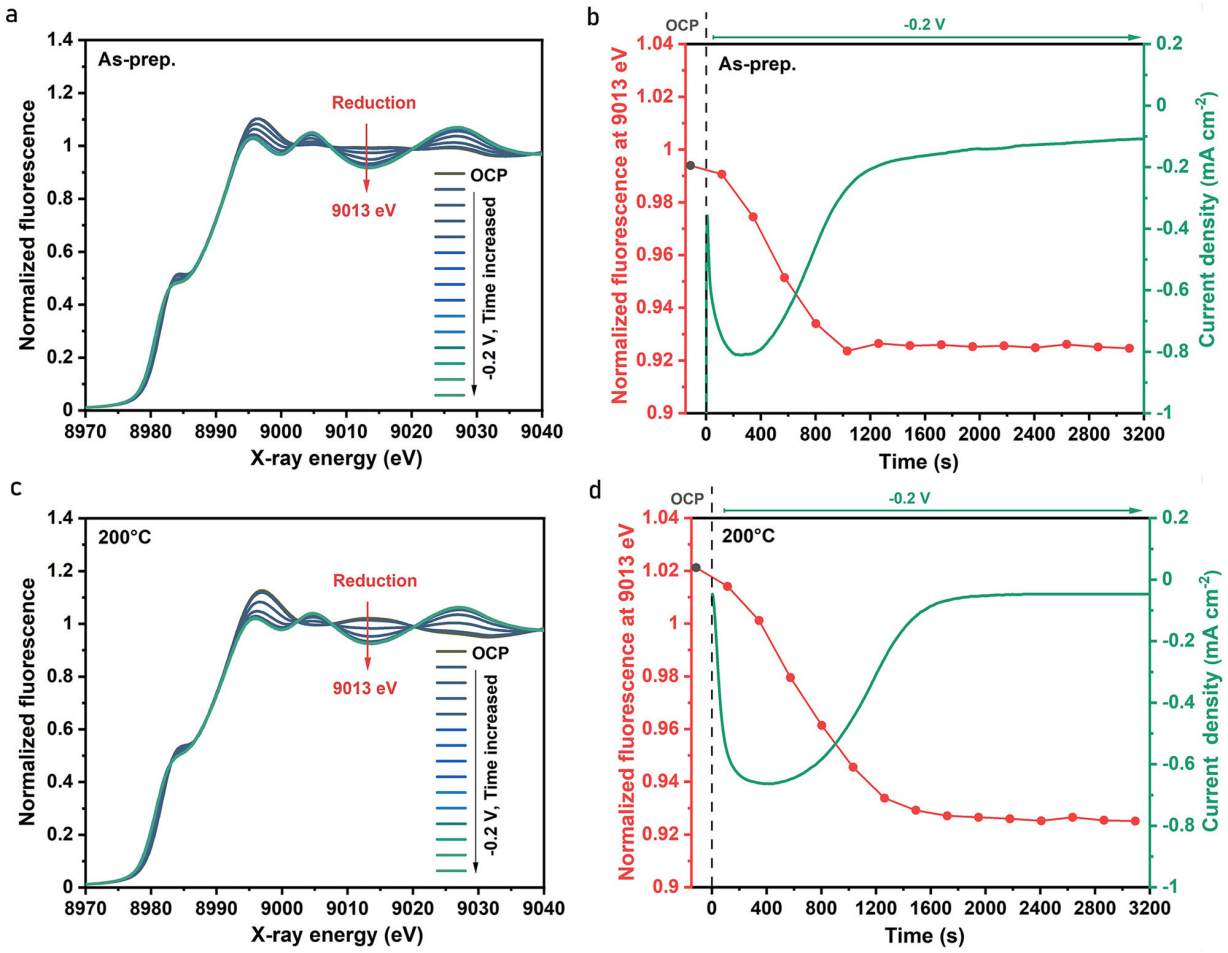
Reduction kinetics of Cu foams from time series of XAS spectra. Data were collected before (at OCP) and during application of an electric potential (−0.2 V) in CO_2_-saturated 0.1 M KHCO_3_ (pH 6.8) on Cu foam (1 A cm^-2^ for 20 s). Time series of Cu K-edge XANES spectra are shown in (**a**) for the as-prepared Cu foam and in (**c**) for the corresponding heat-treated foam. The time courses of the normalized X-ray fluorescence intensity are shown in (**b**) and (**d**) for an X-ray excitation energy of 9013 eV (left y-axes); the respective current densities are displayed on the right y-axes. The time interval between start of subsequent XAS scans was 227 s.

**Fig. 3:**
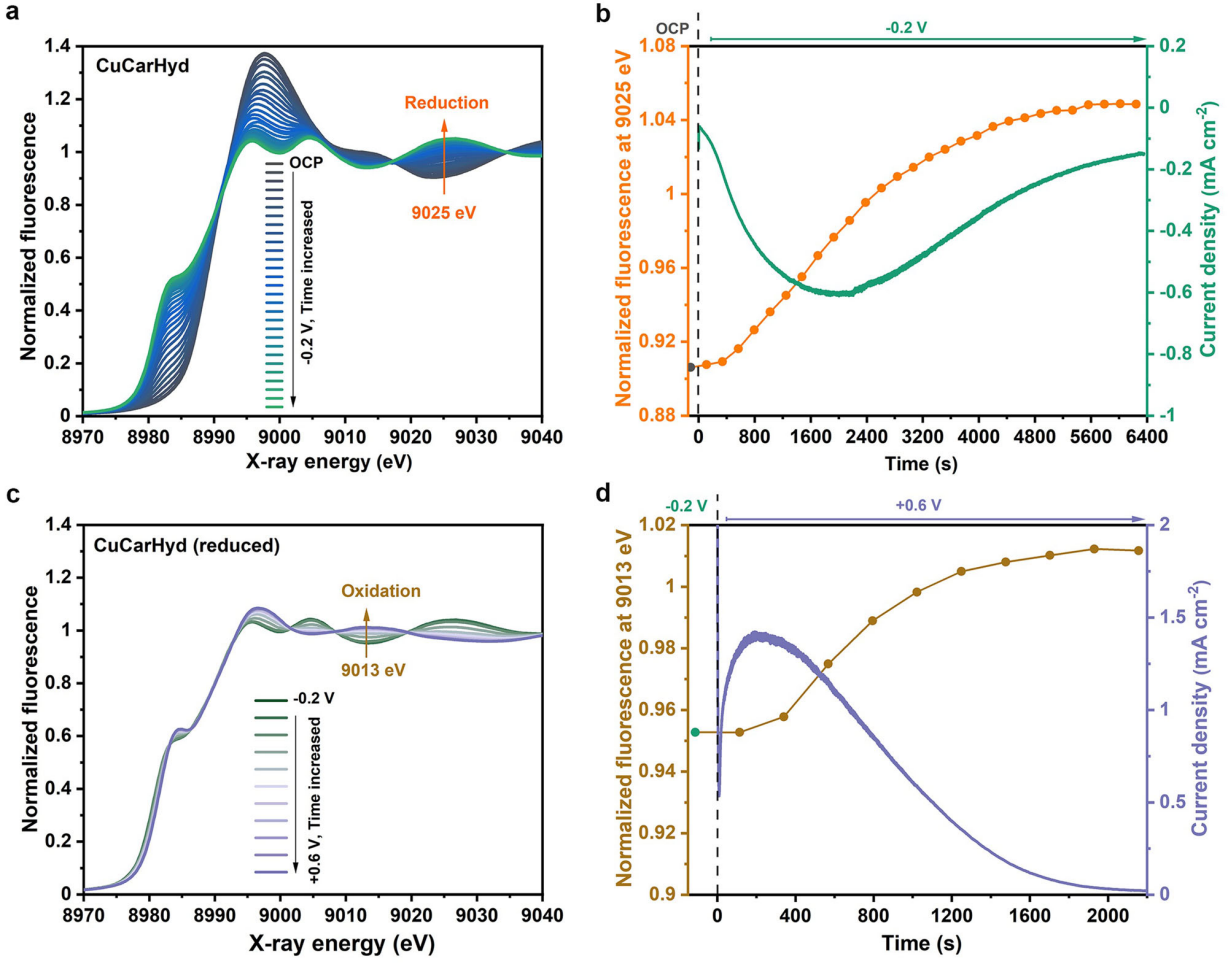
Reduction and reoxidation kinetics of copper carbonate hydroxides (CarCarHyd) from time series of XAS spectra. The data were collected at OCP and during application of an electric potential in CO_2_-saturated 0.1 M KHCO_3_ (pH 6.8). The data of (**a**) and (**b**) relate to reduction at −0.2 V of the Cu foam initially present as CuCarHyd; the data of (**c**) and (**d**) relate to subsequent oxidation at +0.6 V. In (**a**) and (**c**), time series of Cu K-edge XANES spectra are displayed. In (**b**) and (**d**), time courses of current density (right y-axes) and normalized fluorescence intensity (left y-axes) for excitation at 9025 eV/9013 eV are given. The time interval between the start of subsequent XAS scans was 227 s. The reoxidation half-time in (**d**) is 690 s.

**Fig. 4:**
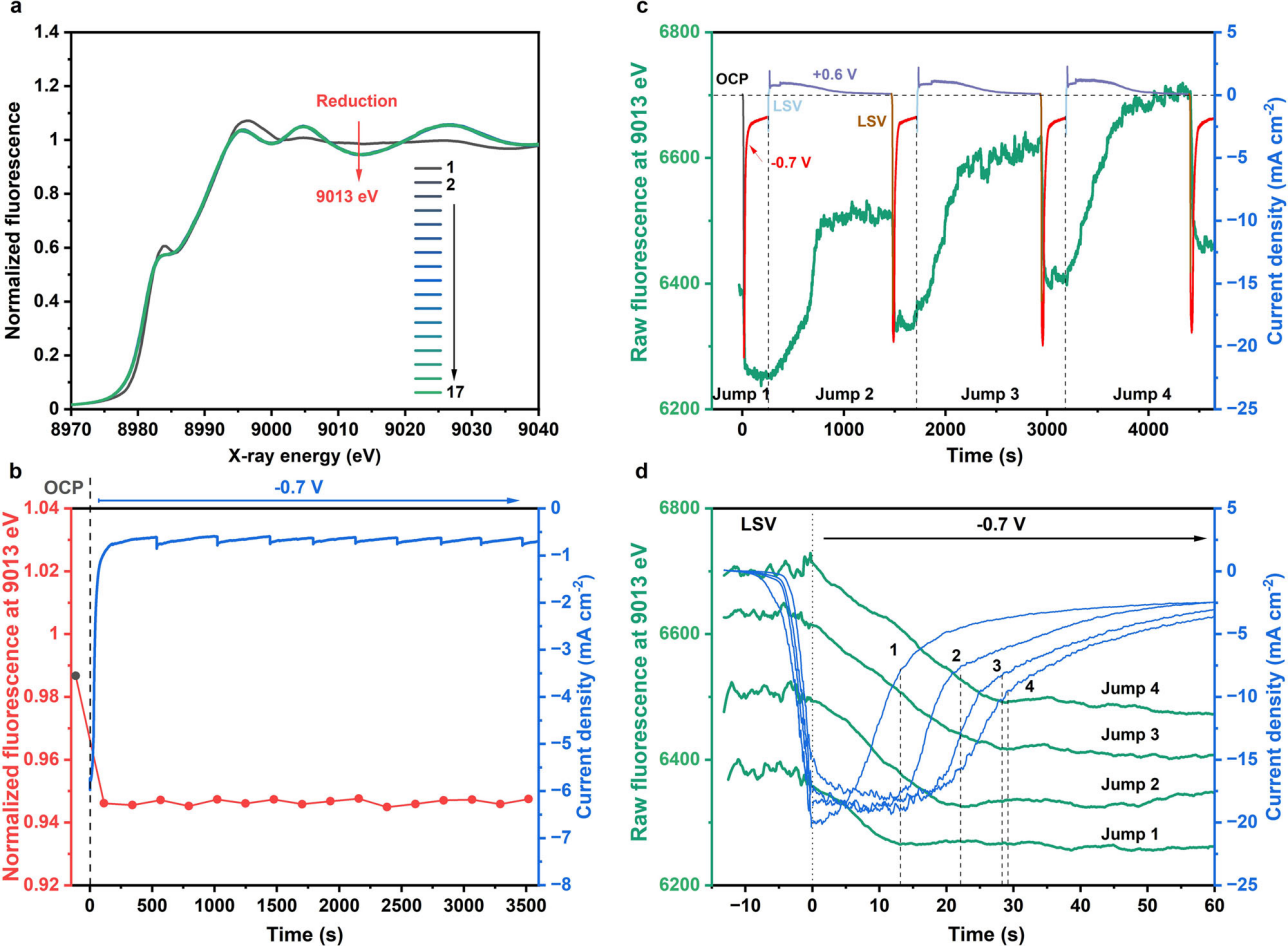
XAS experiments on fast oxide dynamics during potential cycling. Data were collected for the 20s-deposition/air-dried sample at OCP and during application of an electric potential in CO_2_-saturated 0.1 M KHCO_3_ (pH 6.8). **a** Time series of XANES spectra; **b** time courses of normalized X-ray fluorescence for excitation at 9013 eV (left y-axis) and corresponding current density (right y-axis). A total of 17 spectra were collected (for a plot of the stacked spectra, see Supplementary Fig. 15). Spectrum 1 was collected without applied potential (OCP). Spectra 2 to 17 were sequentially collected at −0.7 V with a time interval between spectra of 227 s. Spectra 2-17 are indistinguishable, implying that Cu foam reduction is completed within less than 4 min. In (**c**) and (**d**), raw (not normalized) X-ray fluorescence intensities for excitation at 9013 eV at millisecond time resolution (averaging over 100 ms in the plotted data) are shown. In (**c**), X-ray fluorescence (left y-axis) and current density (right y-axis) time-traces for four +0.6 V (OCP)/−0.7 V potential cycles with non-abrupt transitions between applied potentials facilitated by LSV scans with 100 mV s^−1^ are shown. In (**d**), data as in (**c**), but here shown with an expanded time scale for the four reductive transitions (the time traces of the X-ray signal are labeled by the Jump 1 to Jump 4; the time traces of the corresponding current densities are labeled from 1 to 4). We note that the X-ray data were not corrected for an increase in the incident X-ray intensity during the 1.3 h experiment of Fig. 4c and d, so that the overall increase in the Cu oxidation state may be over-estimated. The redox charges for each reductive and oxidative transition were calculated by integration of the current densities and are shown in Supplementary Fig. 20.

The existence of residual oxides in copper catalysts for CO_2_RR was discussed in earlier studies^14,36^. Cu^+^/Cu^2+^ species near the catalyst surface have even been suggested to be the CO_2_RR active sites^20,37^. To address this topic, we explored whether initial CuO_*x*_ species in the OD-catalysts are susceptible to reduction at moderately negative potentials, tracking related oxidation-state changes with operando XAS. For this, time series of XANES spectra and corresponding electrochemical current densities were recorded on various Cu foams and on CuCarHyd during chronoamperometry (CA) at −0.2 V (Fig. 2, Fig. 3a, b, Supplementary Figs. 7 and 8). The XANES spectra as well as the fluorescence time-traces (at 9013 eV) and corresponding current densities are shown in Fig. 2a, b for the Cu foam sample (20 s deposition time). Simultaneously increasing or decreasing XANES and post-edge features reflect gradual transformation of spectra towards metallic copper, clearly indicating a reduction of CuO_*x*_ species that was completed after about 20 min both in the as-prepared and heat-treated foams (Fig. 2c, d). The existence of several isosbestic points across the XANES spectra indicate a classical two-phase behavior in all examined oxide-to-metal transformations, meaning a transition between two states (phases) of the material without any spectroscopically resolved intermediate (a similar transition has been observed before^38^). Notably, the reduction half-time slightly increases with increasing deposition time despite decreasing relative amounts of oxidic species in the thicker Cu foams, pointing towards slightly increasing absolute amounts in those samples (initial relative oxide contents and reduction half-times are summarized in Supplementary Table 1). As expected, the heat-treated samples, which contain higher relative amounts of oxidic species, cause larger reduction half-times as well. Similar reduction trends were also observed for CuCarHyd (Fig. 3a, b), but the Cu^0^ formation was significantly slower, being completed only after ca. 2 h, possibly relating to the initial presence of Cu^2+^ species (versus predominant contribution of Cu^+^ in the Cu oxide foams). The oxide-to-metal transformation occurs significantly faster at −0.7 V (around 15 s, as later shown in Fig.4d) compared to -0.2 V (20 min) on the same Cu catalyst. Lin et al. ^39^. reported a transformation of 360 s for OD-Cu nanotubes at −0.75 V. Oxygen plasma-activated Cu catalysts^13^ showed Cu and Cu_2_O features after 15 min at −1.2 V, becoming predominantly metallic Cu after 1 h. Eilert et al. ^19^. observed a slow reduction on cubic copper over 20 min at 0.35 V. The faster transformation in our study is highly likely due to the porous foam structure compared to the solid structure of Cu catalysts in the literature.

Interestingly, small but systematic differences of the reduced samples are observed in the XANES spectra (Supplementary Fig. 9), suggesting that – although the reduction process apparently stopped after specific times, depending on the sample thickness (longer deposition time represents thicker sample) and sample history (air-dried Cu foams versus CuCarHyd) – the reduction process in some samples might not be completed in a sense of complete chemical transformation of all oxide species to metallic copper, thus giving an indication for potential kinetic trapping of subsurface/bulk oxide species^40,41^. It should be noted, however, that X-ray flattening effects in the thicker samples may cause similar effects to the XANES spectra, damping the EXAFS-oscillations, and if both were present, deconvolution is not trivial. As an attempt, ex-situ XAS measurements using the transmission mode were conducted at 20 K on Cu foams with varying deposition times. XANES and EXAFS spectra of Cu foams with and without flattening correction are shown in Supplementary Fig. 10. Despite the influence of temperature on the XAS spectra (Supplementary Fig. 11), we can still conclude that flattening effect is minor for the 10 s sample but becomes pronounced for the 30 s sample.

In conclusion, the operando XAS data reveals the presence of a significant amount of copper oxides in the initial Cu foams and CuCarHyd, which can be largely or for thin samples even completely reduced to metallic copper within a minutes-to-hours timeframe and without forming any other intermediates during the reduction process (Supplementary Fig. 12). The potential presence of an oxide layer buried beneath a reduced copper foam is further explored through the following reduction-oxidation schemes.

#### Reoxidation of Cu catalysts at positive potential (+0.6 V)

To study the reoxidation of previously electrochemically reduced Cu catalysts, a potential of +0.6 V was subsequently applied. For this, a Cu foam deposited at 1 A cm^−2^ for 20 s which was air-dried after deposition is compared to CuCarHyd, the latter being obtained by exposure of the electrodeposited Cu foam to carbonate solution for one week. The respective XANES spectra are displayed in Supplementary Fig. 13a, showing that after reoxidation of previously reduced (at −0.2 V) Cu foam and CuCarHyd, similar spectral features as for Cu_2_O emerged for both sample types; linear combinations of reference spectra (Supplementary Fig. 13b) suggest that the reoxidized Cu foam and CuCarHyd have a similar Cu_2_O content (around 60%). However, visual inspection of Supplementary Fig. 13a also reveals non-negligible differences between Cu foam and CuCarHyd in both electrochemical states (reduced and reoxidized), see also Supplementary Fig. 9. For both materials, the reduction and oxidation processes were almost complete, so the same spectra would be expected for both sample types, unless the starting material exerts a memory effect that shapes the material structures subsequently formed by reduction and reoxidation. Therefore, we tentatively conclude that the structures formed by completed electrochemical reduction and reoxidation of the Cu foam material depend on the history of the preceding redox chemistry.

The kinetics of the oxidation process of previously reduced Cu foam and CuCarHyd are detailed in Supplementary Fig. 14 and Fig. 3c and d, respectively. Cu^2+^ species are not formed during the oxidation of both samples, but the time required for the oxidation of the reduced Cu foam (half-time: 1030 s) is about 1.5-fold longer than that for the oxidation of the reduced CuCarHyd, which could be explained by the memory effect proposed above for the originally synthesized material. The reoxidation also follows a two-phase transformation (transition between two states/phases of the material without intermediate state/phase), as indicated by distinct isosbestic points in the XANES spectra.

Upon analyzing the reduction and oxidation process data (Figs. 2 and 3 and Supplementary Figs. 7 and 8 and 14), we consistently observed in the X-ray signal an initial delay (some minutes) in the redox-state changes of the Cu foams and CuCarHyd, which correspond to an initial rise in the (absolute value) of the current density towards a peak value which is reached several minutes after onset of the potential change. This induction phenomenon can possibly be explained by a passivating water-overlayer covering the oxide and/or by the requirement of an initial formation of promoting structures before the redox state changes of the bulk material can start.

#### Cycling between oxidative and catalytic potential

To study the redox dynamics of the OD-Cu catalyst when exposed to catalytic potentials, we performed time-resolved XAS experiments at −0.7 V. As shown in Fig. 4a, b and Supplementary Fig. 15, the transformation of the Cu foam to metallic copper appears to be complete after collection of the first XANES spectrum (around 4 min). Already the second spectrum resembles the last spectrum collected after ca. 1 h. Interestingly, the lag-phase observed at −0.2 V and +0.6 V is absent at −0.7 V for both Cu foam and CuCarHyd (Supplementary Fig. 16). Moreover, a considerable difference is observed in the XANES spectra of samples reduced at moderate −0.2 V or catalytic −0.7 V (Supplementary Fig. 17), clearly indicating that the reduction process at −0.7 V ends faster but is nevertheless less complete, suggesting the quick formation of a metallic layer in the surface-region, thereby kinetically hindering oxide reduction in the subsurface/bulk parts. This can be tracked by the different values of the fluorescence intensity at characteristic values (e.g. at 9013 eV, Supplementary Table 2). Taking the thinnest sample deposited at 10 s as reference, the expected value would be about 0.98 and 1.02 for the as-prepared oxidized species and about 0.92 for a basically completely reduced sample. Slightly larger values of about 0.93-0.94 are found for the thicker Cu foams and CuCarHyd reduced at −0.2 V, suggesting incomplete reduction processes as discussed above. For the samples reduced at −0.7 V, the fluorescence intensity is about 0.95, supporting the hypothesis. When comparing Cu foams reduced under various conditions (Supplementary Fig. 18), including both 20 K and 293 K measurements, and considering EXAFS flattening or not, a consistent minimum value at 9013 eV at approximately 0.92 is observed. This outcome further supports the assertion that the 10 s sample in its reduced state is the most extensively reduced among all the samples.

As discussed above, conventional XAS operates on a timescale of minutes for collection of a spectrum, while the oxidation state of the Cu centers could reach a constant level within less than 4 min at −0.7 V (Fig. 4a, b). Consequently, we aimed at tracing oxidation state changes of Cu on a time scale of seconds. To this end, CA experiments were performed at selected potentials and X-ray fluorescence intensity changes during the voltage jumps were monitored at a fixed excitation energy (9013 eV) with a time resolution of 1 ms per data point^42,43^. We used a potential step sequence, in which the cathode potential was held at −0.7 V for a time period of 4 min and was then stepped to an anodic potential of +0.6 V for 20 min. For sudden jumps between these potentials, we detected sample degradation in the spectra and as visible loss of small fragments of the Cu material. To prevent strain from too abrupt material transformation, linear sweep voltammetry (LSV) with a scan rate of 100 mV s^−1^ was applied between the potential steps, thereby largely preventing catalyst degradation. Respective XAS spectra of Cu foam before and after the potential jumps collected under OCP condition (Supplementary Fig. 19a, b) confirm the absence of significant catalyst degradation.

The X-ray fluorescence and current density time-traces during the CA steps are shown in Fig. 4c. The potential-dependent fluorescence changes at 9013 eV track the oxidation state changes of the Cu material. Figure 4d and Supplementary Fig. 19c, d, show that the fast LSV has a negligible effect on the fluorescence intensity at 9013 eV, suggesting that the interlaced LSVs are largely innocent with respect to the targeted kinetics of oxidation and reduction of the Cu material. The reduction process at −0.7 V is also shown in Fig. 4d (time-extended data is given in Supplementary Fig. 19c). It takes around 12 s to reduce CuO_*x*_ in potential jump 1, thus being about 80 times faster than the reduction process at −0.2 V (Fig. 2b). The pronounced potential dependence of the reduction rate suggests that rather than electrolyte proton transport or other mass transport limitations, the catalyst-internal redox chemistry limits the reduction rate at low potentials. This is in accordance with the absence of a lag-phase for the reduction process at more negative potentials.

**Fig. 5:**
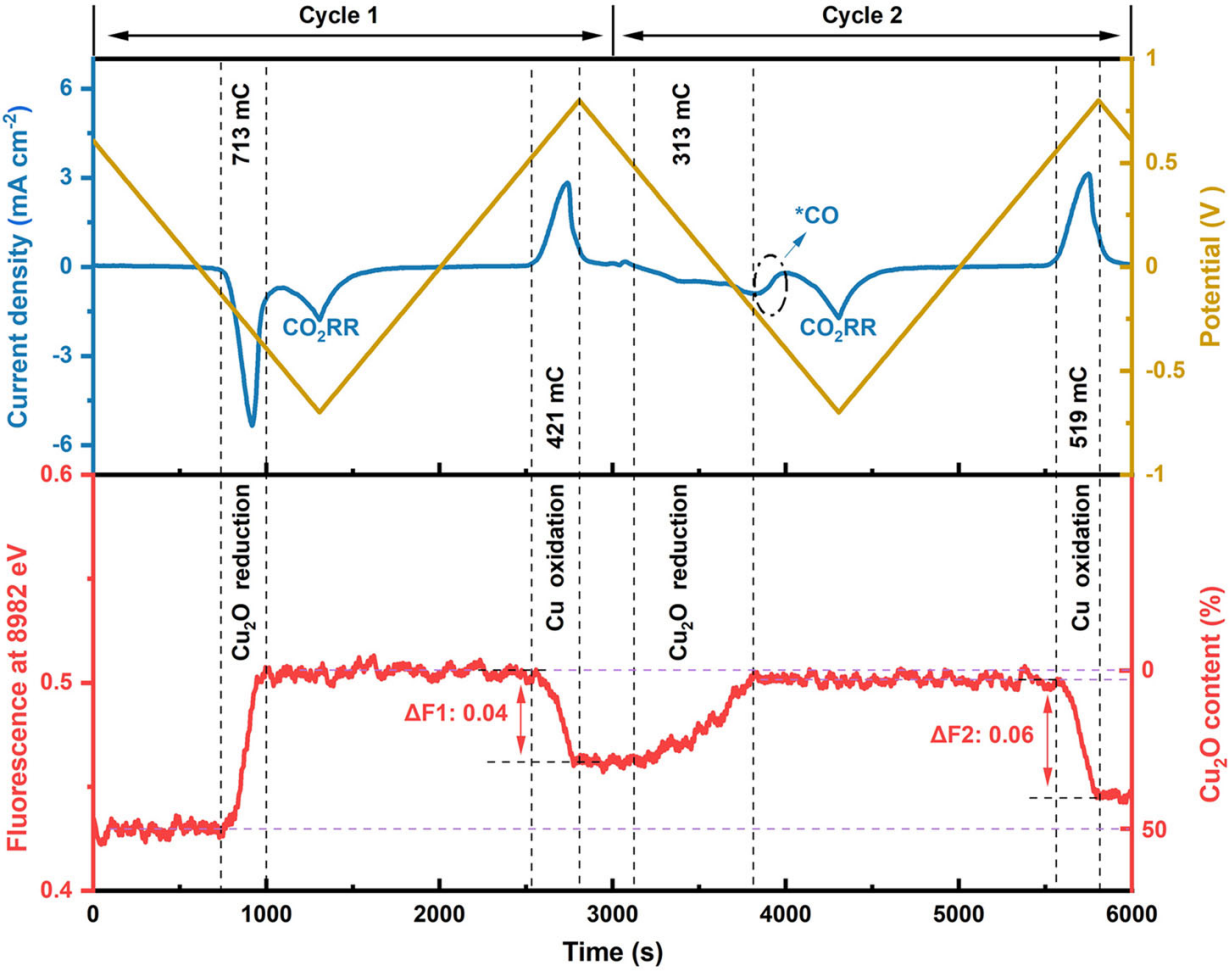
X-ray fluorescence time traces during CV cycles. Data were collected for the 20s-deposition/air-dried sample in CO_2_-saturated 0.1 M KHCO_3_ (pH 6.8) during two CV cycles (CV start at OCP; forward scan down to −0.7 V, backward scan to +0.8 V, 1 mV s^−1^). Detection of the CV current was combined with recording of the X-ray fluorescence at four excitation energies; here the normalized 8982 eV X-ray fluorescence intensity is shown. Top panel: potential (yellow line) and current (blue line) time traces. Bottom panel: normalized X-ray fluorescence time-trace (red line). The Cu_2_O content is taken from the linear combination of XANES spectra in Supplementary Fig. 5c. ΔF1 and ΔF2 represent the fluorescence changes during oxidation process for cycle 1 and cycle 2, respectively. The normalized process for fluorescence intensity and the calculation of integrated charge for oxidation and reduction are described in section 2.3 of the Supplementary Information (SI). The integration ranges were chosen such that outside the integration range the X-ray signal was constant, thereby verifying the absence of redox-state changes of the Cu material outside the indicated integration ranges.

Figure 4d reveals that the reduction period increases with the increasing number of reduction-oxidation cycles from about 12 s in Jump 1 to about 30 s in Jump 4, which correlates with an increase in the respective reduction charge obtained by current integration (Supplementary Fig. 20). We conclude from Fig.4c, d and the Supplementary Figs. 19 and 20 that repeated reduction-oxidation cycles result in (i) an increase in the magnitude of oxidation-state changes for both, application of reductive and oxidative potential and (ii) an increasingly oxidized Cu-foam at reducing potentials. The above observations are remarkable because they suggest as a consequence of repeated potential cycling (i) an increase in the susceptibility of the Cu material to redox-state changes and (ii) an accumulation of more Cu^+^ species. Both suppositions are likely relevant regarding the presence of subsurface oxide at reducing potentials^20,41,44–46^ and the mechanisms of modifying selectivity and stabilizing CO_2_RR activities by potential cycling^32,47–49^. However, the experiment of Fig. 4 may not be fully conclusive because of ambiguities possibly introduced by the slow oxidation phases, which could have hindered reaching a steady-state level at the respective potential. Yet the combination of cyclic voltammetry and operando XAS (Fig. 5) supports both above conclusions, as discussed in the following.

#### Oxide formation during cyclic voltammetry

CV and XAS data collection were combined to study the potential-dependent oxide formation. As shown in Supplementary Fig. 21a, the X-ray fluorescence intensities for excitation at 8960 eV (pre-edge background level) and 9020 eV (post-edge background level) are unaltered by the reduction process, thereby providing suitable reference points for normalization of the 8982 eV and 9013 eV X-ray intensities, which were used to track Cu oxidation-state changes. The respective X-ray fluorescence intensities at these four energies were recorded during 2 CV cycles (1 mV s^−1^, Supplementary Fig. 21b). The normalized 8982 eV X-ray intensity is displayed in Fig. 5, where an increase (decrease) in the X-ray fluorescence at 8982 eV indicates a decrease (increase) in the Cu oxidation state. The fluorescence changes at 8982 eV and 9013 eV show similar trends but opposite directions of changes (see Supplementary Fig. 21c–f).

For the first CV cycle, the fluorescence intensity increases strongly between −0.2 V to −0.4 V in the forward scan, showing a reduction of the Cu foam. The intensity decreases from +0.5 V to +0.7 V in the backward scan, indicating that the Cu foam is reoxidized. For Cycle 2, the intensity already starts to increase at around +0.5 V (versus −0.2 V in Cycle 1), indicating specific kinetics for reduction of the electrochemically formed oxide. In the potential range of −0.2 V to −0.4 V, a current wave is observed without any accompanying fluorescence changes. This phenomenon is consistent with previous studies^35,50^ and can be attributed to CO_2_ or carbonate reduction^22^ resulting in CO adsorbed at the catalyst surface.

Figure 5 shows for both CV cycles that after reduction of the material a plateau level of the X-ray signal is reached, which verifies that the oxidation state of the Cu foam has reached a steady-state. We note that the fluorescence intensity at the steady-state for Cycle 1 is slightly higher than that for Cycle 2, implying that a less reduced (more oxidized) Cu foam is formed by reduction in Cycle 2. This is qualitatively in line with the integrated charge for Cu oxidation in Cycle 1 (421 mC) exceeding that for CuO_*x*_ reduction in Cycle 2 (313 mC). We conclude that in Cycle 2 likely a more oxidized Cu foam was formed than in Cycle 1, even though also in Cycle 1 a steady-state level had been reached, but at a slightly lower value. Moreover, we find larger changes in the second electrochemical oxidation, thus a clearly more oxidized state is reached than in the first one (ΔF_1_ > ΔF_2_), in line with the corresponding redox charges. These findings support the conclusions from the potential-cycling experiment of Fig. 4, namely that repeated potential cycling causes (i) an increase in the susceptibility of the Cu material to redox-state changes and (ii) the accumulation of more Cu^+^ oxide species. We assume that the Cu oxide species that are kinetically stable at negative potentials (on the investigated time scale) here detected by bulk-sensitive operando-XAS relate to the subsurface oxide observed using near-surface sensitive techniques^20,40,51^. In the following, the dynamics of chemical species at the Cu foam surfaces is investigated by operando Raman spectroscopy, exploiting that the Cu foam material exhibits an intrinsic surface-enhancement effect^23^.

### Operando Raman spectroscopy

#### Surface oxide reduction and electrochemical re-formation

**Fig. 6:**
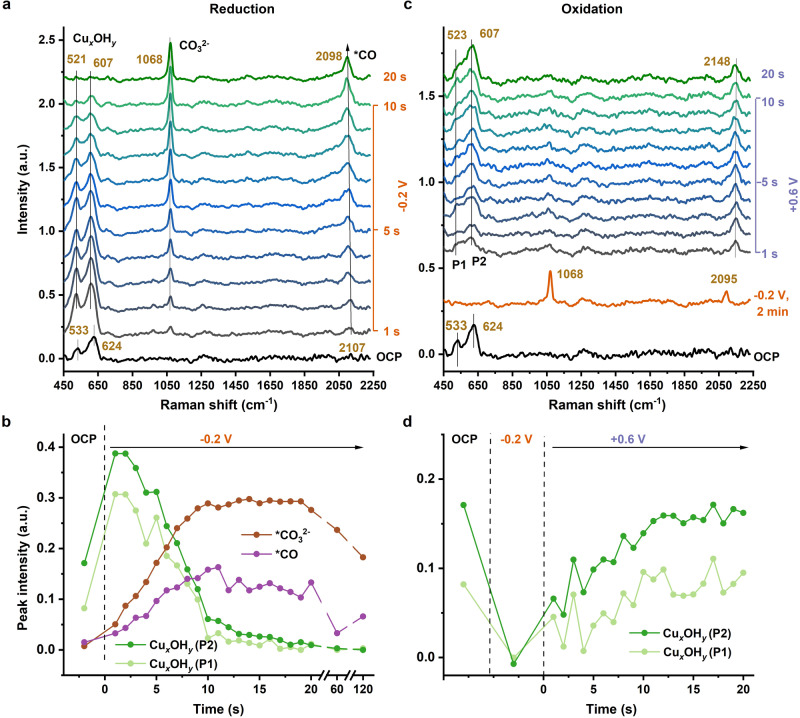
Surface oxides reduction (at -0.2 V) and electrochemical reformation tracked by time series of Raman spectra. Data were collected at OCP and during application of an electric potential in CO_2_-saturated 0.1 M KHCO_3_ (pH 6.8). Time-series of SERS spectra of Cu foam were recorded at (**a**) −0.2 V and (**c**) +0.6 V. Time-courses of band intensities of indicated species are shown in (**b**) and (**d**). For presentation of (**a**) and (**c**) with spectra collected at additional times and the assignment of further bands, see Supplementary Fig. 22. The same Cu-foam sample was used in (**a**) and (**c**) and was measured at OCP condition firstly before being exposed to reducing (−0.2 V) and oxidizing (+0.6 V) potential. The procedure for determination of the maximum peak intensity is described in Section 3 of the SI.

Surface-sensitive operando Raman spectroscopy facilitated by the intrinsic SERS activity of the Cu foam material^23^ was used to probe the potential- and time-dependent appearance of various species at the Cu foam surface. The formation or decay of surface oxide populations (and of other species) was monitored by collecting time series of spectra (time-spacing of 1 s) at potentials of -0.2 V or +0.6 V (Fig. 6, Supplementary Fig. 22). Two broad bands in the region of 450–650 cm^−1^ visible at OCP condition stem from Cu oxides/hydroxides (Fig. 6a, labeled as Cu_*x*_OH_*y*_)^23^. Comparison with spectra of Cu reference compounds (Supplementary Fig. 23) suggests that the oxides in as-prepared Cu foams are a mixture of Cu_2_O and small amounts of Cu_2_CO_3_(OH)_2_. Switching the potential from OCP to −0.2 V, the Cu_*x*_OH_*y*_ bands shift immediately to lower wavenumbers (533 to 521 cm^−1^ and 624 to 607 cm^−1^), presumably explainable by an electrochromic shift of the band positions resulting from the applied negative potential. These surface oxide bands essentially disappear largely within only about 12 s (Fig. 6b), which compares to about 1000 s for reduction of the bulk oxides (Fig. 2b) at this low negative potential, suggesting rapid formation of a metallic overlayer covering a material-intrinsic oxide phase.

A dominant feature in the spectra at −0.2 V is a band centered at 1068 cm^−1^, originating from the symmetric C-O stretching mode of carbonate (*CO_3_^2-^), with essentially time-independent band position (Supplementary Fig. 22a). A broad band in the region of 1950–2150 cm^−1^ is assignable to the CO stretching mode (*CO)^22,23,52–54^. This *CO peak displays a red shift over time at −0.2 V (2107 to 2098 cm^−1^, see also Supplementary Fig. 24a), with the band shift being likely of electrochromic origin, in line with previous study^55^. The *CO_3_^2−^ and *CO intensities increase within the first 10 s and gradually decrease thereafter (Fig. 6b). Supplementary Fig. 24b displays the peak intensities of *CO and *CO_3_^2−^ plotted against the peak intensity of the Cu_*x*_OH_*y*_ (P2) band. When the peak intensity of Cu_*x*_OH_*y*_ approaches zero, the *CO and *CO_3_^2−^ bands do not show any further increase and even exhibit a slight decrease, suggesting that the disappearance of oxides and the adsorption of *CO and *CO_3_^2−^ are not directly correlated events. We conclude that binding-site blockage by surface oxides cannot straightforwardly explain the *CO and *CO_3_^2−^ adsorbate kinetics.

Reoxidation of the Cu foam at +0.6 V was monitored by SERS (Fig. 6c). The as-prepared Cu foam was reduced at −0.2 V for 2 min (see Fig. 6a) and then the potential was switched to +0.6 V oxidizing potential. As expected, the Cu_*x*_OH_*y*_ bands are the main features in the spectra collected at +0.6 V and the intensity of these bands increases over time (Fig. 6d). This indicates that metallic Cu is gradually oxidized to Cu_*x*_OH_*y*_ until after ca. 12 s a steady state is reached. We also discover a new band at 2148 cm^−1^ that rises in parallel to surface oxide formation. Its vibrational frequency points towards a CO band. To identify the origin of this presently band requires further investigations.

Combining the results obtained by operando-XAS for the bulk material and operando-SERS for surface species implies that in the reduction process initially a metallic layer is formed covering a mixed oxidic/metallic phase in the bulk material. Conversely, in the electrochemical oxidation process, rapid oxidation of the metallic surface proceeds with fast kinetics that are unrelated to the slow oxidation of the bulk material.

#### Surface species developing at CO_2_RR potential (−0.7 V)

**Fig. 7:**
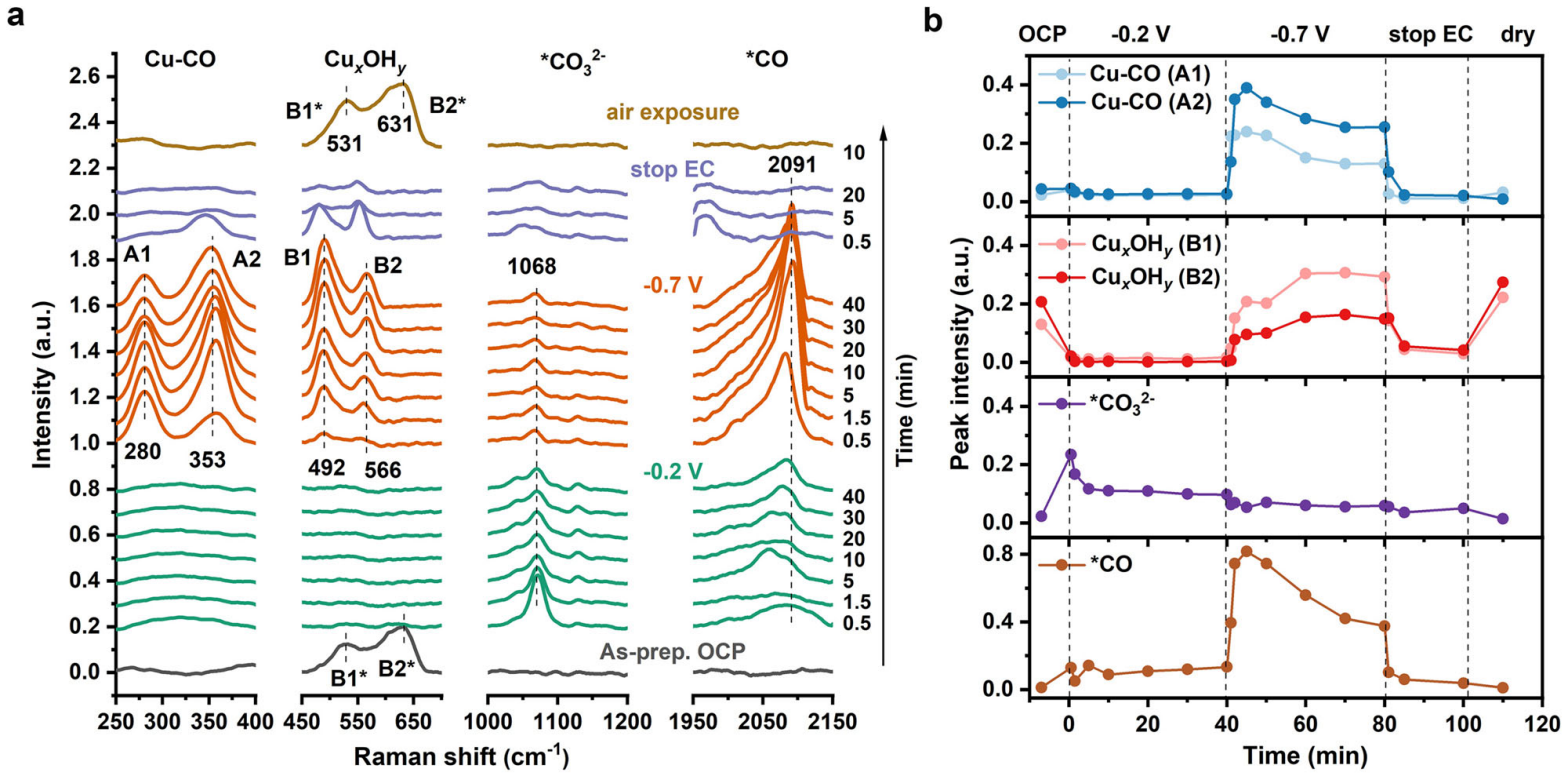
Operando Raman spectra of Cu foam. Data were collected at OCP and during application of the indicated electric potentials in CO_2_-saturated 0.1 M KHCO_3_ (pH 6.8). **a** Raman spectra split into four sections to highlight the Cu–CO, Cu_*x*_OH_*y*_ and adsorbed *CO_3_^2-^, *CO bands (extended spectral range in Supplementary Fig. 25a). Gray: OCP; green: −0.2 V for 40 min; orange: −0.7 V for 40 min; purple: “stop EC” for 20 min (no potential applied, OCP condition); dark yellow: air-exposed Cu foam in the “dry” state. **b** Time-traces of Cu–CO (A1, A2), Cu_*x*_OH_*y*_ (B1, B2 or B1*, B2*), adsorbed *CO_3_^2-^ and *CO bands intensities. The displayed results here are the average of two datasets (as shown in Supplementary Fig. 26).

To investigate the evolution of surface species during CO_2_RR, long-term SERS measurements were performed (Fig. 7a; for complete spectra see Supplementary Fig. 25a). At −0.7 V, the bands located at 280 cm^−1^ and 353 cm^−1^ are assigned to the restricted rotation of adsorbed CO and Cu–CO stretching modes (A1 and A2 bands), respectively^56^. Of particular interest are the bands at 492 cm^−1^ and 566 cm^−1^ (B1, B2) in the 450–650 cm^−1^ range, which are possibly related to Cu hydroxides, but clearly differ from the B1*/B2* bands in the as-prepared Cu foam.

The time dependence of the Cu–CO, Cu_*x*_OH_*y*_, *CO_3_^2-^ and *CO band intensities is shown in Fig. 7b. At −0.7 V, the Cu–CO bands and *CO band concurrently and significantly increase in the first five minutes then decrease in the next 35 minutes. This behavior mainly reflects the balance of the CO generation with CO desorption and/or by conversion from CO to hydrocarbons at −0.7 V. Interestingly, the time-dependent behavior of the intensity ratio of A2/A1 (Supplementary Fig. S25b) aligns with the trend observed in the B1 and B2 bands, that is rising towards saturation. The A2/A1 ratio is commonly used as a measure of the surface CO coverage, with a higher ratio associated with a higher FE for C_2+_ products^56,57^. The pathways of C–C coupling are dependent on factors such as catalysts properties, electrolyte composition, local pH and electrochemical protocols^58–61^. Accordingly, they encompass diverse possibilities, including *CO–CO, *COH–CHO, *COH–*COH, and *CO–*HCHO, among others. Given the complexity of the CO_2_RR system, deconvoluting the specific C–C coupling pathway is challenging. We could tentatively conclude that the formation of new species being reflected by the B1 and B2 bands promotes the increased *CO coverage, increasing the possibility for CO dimerization, and is a material property that grows only after exposure to catalytic potentials for tens of minutes.

In conclusion, the XAS and SERS data reveal that at −0.7 V, the CuO_*x*_/Cu_*x*_OH_*y*_ species are rapidly reduced both at the surface and within the bulk of the copper catalysts, however, not completely in the bulk (see discussion above). The Raman bands in the 450–650 cm^−1^ region are formed during CO_2_RR at −0.7 V rather than being retained from the initial Cu oxides. The slow formation of these bands correlates with a change of the *CO binding mode (increased amplitude ratio of A1 and A2 peak), which presumably relates to reaction paths in CO_2_RR that favor formation of C_2+_ products^56,62^. Vibrational bands possibly assignable to hydroxide formation at catalytic potentials are formed at catalytic potential in the 450–650 cm^−1^ region. Their further characterization is approached in the following.

#### Vibrational bands in the 450–650 cm^−1^ region formed at CO_2_RR potential

**Fig. 8:**
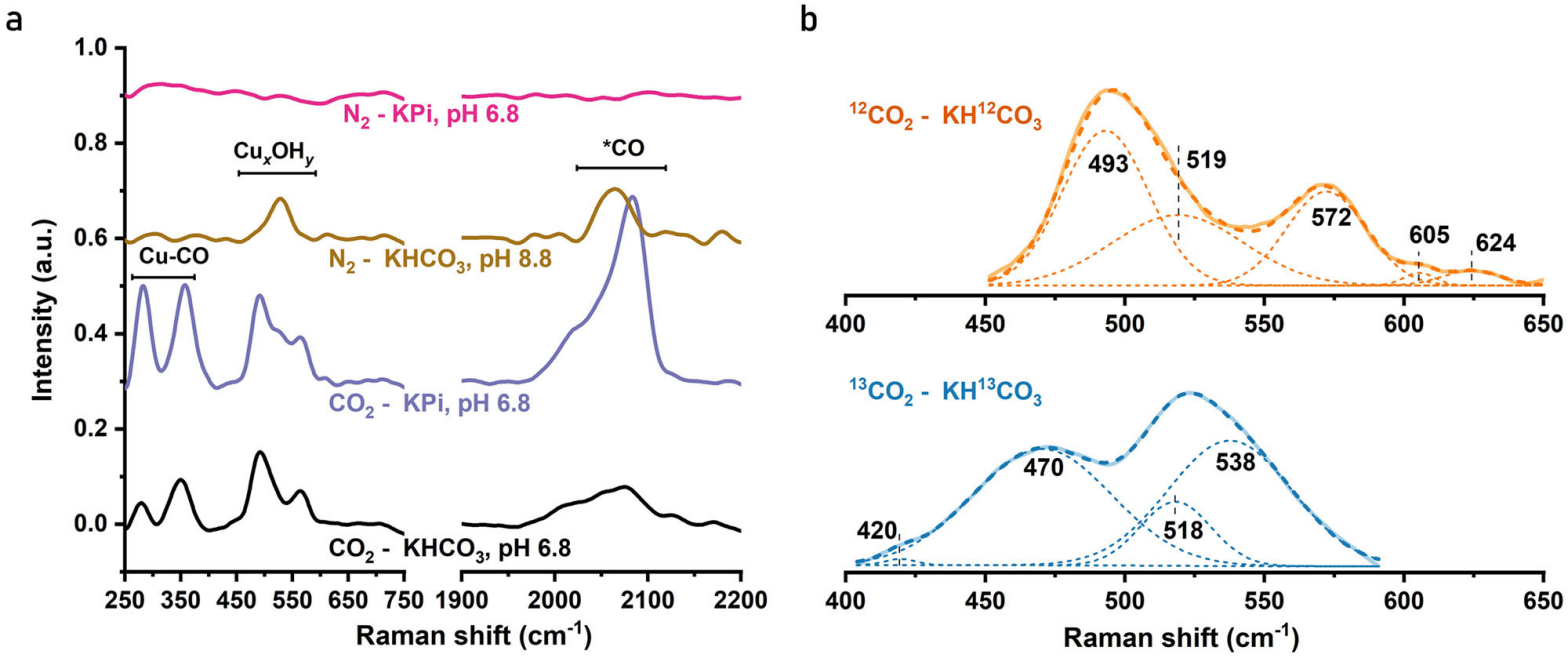
Operando SERS spectra of Cu foam in different electrolytes. The Cu foams were operated at -0.7 V for 30 min. **a** Representative spectra of Cu foam in CO_2_-saturated KHCO_3_ (black line), CO_2_-saturated KPi (purple line), N_2_-saturated KHCO_3_ (brown line) and N_2_-saturated KPi (magenta line) electrolytes (0.1 M). **b** The spectrum obtained in ^13^CO_2_-saturated KH^13^CO_3_ (0.1 M) can be described by four Gaussian components (blue line) and the spectrum obtained in ^12^CO_2_-saturated KH^12^CO_3_ (0.1 M) by five Gaussian components (orange line). Experimental data, solid lines; simulated curves, dashed lines.

The SERS experiment was carried out at catalytic potential of -0.7 V not only under the standard conditions of CO_2_-saturated 0.1 M KHCO_3_ electrolyte (pH 6.8) but also in N_2_-saturated potassium phosphate (0.1 M KPi), CO_2_-saturated KPi, and N_2_-saturated KHCO_3_ (at pH 8.8), see Fig. 8a and Supplementary Figs. 27–29. As clearly visible in Fig. 8a, in N_2_-saturated KPi electrolyte (magenta line; extended spectral range in Supplementary Fig. 29b), neither *CO bands nor the bands in the 450–650 cm^−1^ region can be detectable, whereas in both CO_2_-saturated KPi (purple line) and KHCO_3_ electrolyte (black line) these band are well resolved, supporting an intimate relation to CO_2_ reduction of the 450–650 cm^−1^ bands and pointing towards a possible contribution of carbon species to these vibrational bands.

For the spectra collected in N_2_-saturated KHCO_3_ (Fig. 8a, brown color; extended spectral range in Supplementary Fig. 29a), in line with a previous study^56^, Cu–CO bands are either fully absent or present at only negligible intensities. Notably, a single band at 524 cm^−1^ in the Cu_*x*_OH_*y*_ region remains consistently detectable, indicating its independence from CO_2_ saturation. For N_2_-saturated KHCO_3_ electrolyte, the pH of the system increases from 6.8 to 8.8, likely promoting the formation of hydroxides. This supports the assignment of the 524 cm^−1^ band to Cu–OH vibrations.

#### ^12^C/^13^C isotope exchange

To shed more light on the identity of the 450–650 cm^−1^ bands, ^13^C/^12^C exchange experiments were carried out. The interesting region of the representative spectra for ^12^CO_2_-saturated KH^12^CO_3_ or ^13^CO_2_-saturated KH^13^CO_3_ electrolytes are shown in Fig. 8b (extended spectra in Supplementary Figs. 30 and 31). Both spectra show three major Gaussian components in the 450–650 cm^−1^ region. The band at 519 cm^−1^ remains unchanged in ^13^C vs. ^12^C electrolyte, and a similar band at 524 cm^−1^ is also present in the N_2_-saturated KHCO_3_ electrolyte (Supplementary Fig. 29a). Referring to the detailed discussion presented in the Supplementary Note 1^18,25,52,63–67^, we assign this band to the Cu–OH vibration. In contrast, the bands at 493 cm^−1^ and 572 cm^−1^ in the ^12^C electrolyte are red-shifted to 470 cm^−1^ and 538 cm^−1^ in the ^13^C electrolyte, which implies that these vibrations involve C atoms (see also Supplementary Fig. 30). As discussed in detail in Supplementary Note 2 and in agreement with previous studies^18,25,52,63–67^ (Supplementary Table 3), the 493 cm^−1^ band is assigned to Cu-(CX) vibration. The 572 cm^−1^ band, which is influenced by ^13^C/^12^C and N_2_/CO_2_ exchange, does not align with the characteristic peaks of the Cu hydroxy carbonate reference (Supplementary Fig. 28b). Therefore, it is plausible to attribute the 572 cm^−1^ band to a vibration mode of C-containing intermediates formed during CO_2_RR.

In summary, operando SERS analysis suggests the presence of three surface species formed upon prolonged operation at catalytic potential: copper hydroxides (adsorbed *OH on the Cu surface) detected at 519 cm^−1^ as well as one or two C-containing species with characteristic vibrational frequencies of 493 cm^−1^ and 572 cm^−1^. Previous investigations have confirmed the presence of subsurface oxygen in the OD-Cu catalysts using surface-sensitive XPS. However, the potential existence of *OH species has not been observed in these studies due to the inherent constraints of XPS technique^37,40^. By applying operando XAS and Raman we could demonstrate that subsurface oxides and *OH species coexist in the OD-Cu foam even at strongly reductive potentials. Also, the adsorption of *OH and *CO is promoted due to Cu^+^/Cu^2+^ formed by subsurface oxygen within the Cu electrode^68–70^ (more details in Supplementary Note 3).

## Conclusions

The generation and transformation of surface and subsurface/bulk oxide species in OD-Cu catalysts is affecting the product selectivity, in particular by favoring high-value C_2+_ products. In our study on Cu foams, the relevant electrochemical processes, i.e., reduction at moderately negative or catalytic potentials, reoxidation and oxidation/reduction cycles, were investigated by a suitable combination of operando spectroscopic techniques, specifically bulk-sensitive XAS and surface-sensitive Raman methods. The initial catalyst composition, e.g., with respect to the Cu oxide vs. metal contents, strongly depends on the deposition time and post-deposition procedures such as air or carbonate solution exposure, or high-temperature treatment (in air). The bulk oxides in the as-prepared Cu foams are mainly composed of Cu_2_O and its relative amount increases by heat treatment, whereas the surface oxides consist of Cu_2_O with minor Cu_2_(OH)_2_CO_3_ admixtures.

**Fig. 9:**
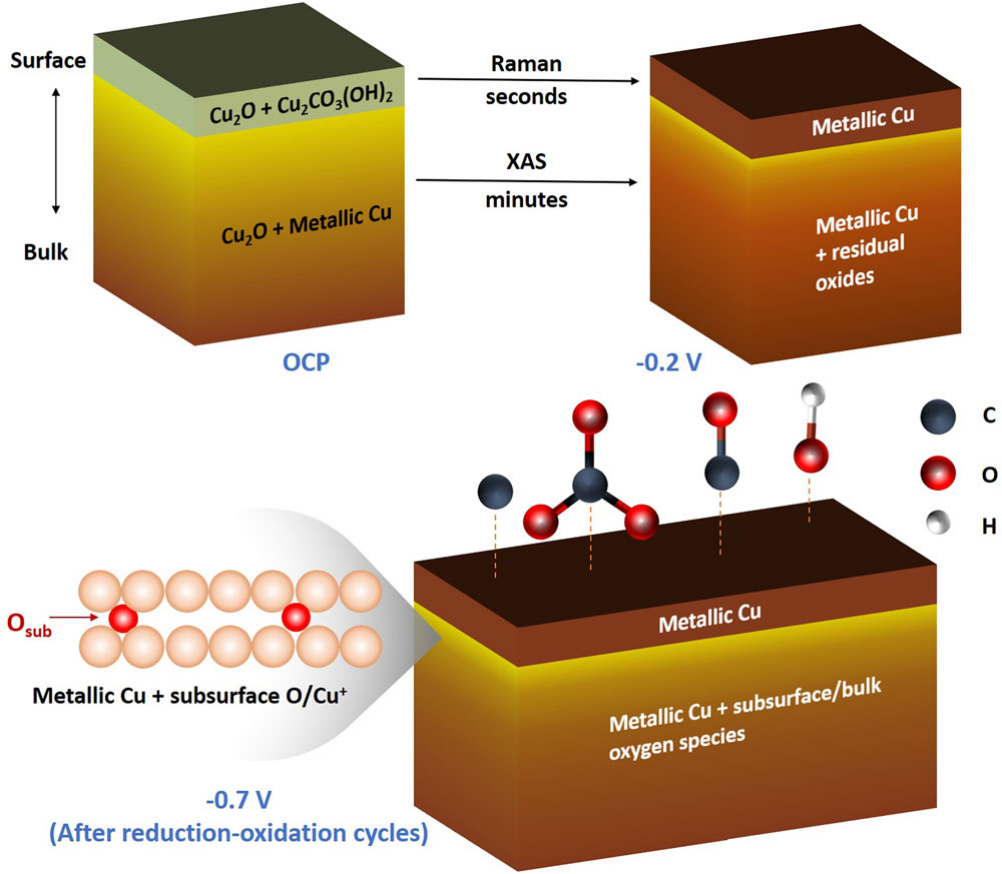
The evolution of chemical species in the surface and bulk of the OD-Cu catalyst under in-situ conditions. At OCP, the OD-Cu material consists of metallic Cu, Cu_2_O and Cu_2_CO_3_(OH)_2_. At −0.2 V (vs. RHE), surface oxides can be reduced in seconds as observed by operando Raman spectroscopy. Bulk oxides can be largely reduced in tens of minutes as observed in operando XAS. At −0.7 V, reduction-oxidation cycles (potential jumps or CVs) have resulted in the formation of subsurface/bulk oxide species; moreover, surface adsorbed C-containing species (*CO_3_^2-^, *CO and *CX) and most likely also hydroxide species (*OH) are detectable.

OD-Cu reduction at a moderately negative potential (−0.2 V) and reoxidation (+0.6 V) typically occur within the minute timeframe (half-times of 500–1000 s), whereas reduction at −0.7 V occurs within seconds, i.e., about 80 times faster. Notably, a lag-phase for Cu oxidation-state changes is observed both at mild reduction condition of −0.2 V and reoxidation steps, which may reflect the requirement for preceding changes in the material that facilitate the subsequent redox reactions. For reduction at catalytic potential (−0.7 V), such a lag-phase was not resolved, which is likely explained by the limited time resolution of the used experimental protocol. Surprisingly, the reduction processes tend to be generally incomplete, that is, the complete conversion to a well-ordered metallic Cu phase is never observed (see also Fig. 9). The extent of metal phase formation depends on Cu foam thickness, the magnitude of applied negative potential and the ‘history’ of previously applied potentials. The incomplete Cu metal formation implies kinetic trapping of subsurface/bulk oxide species. This effect was particularly apparent when gentle reduction conditions were compared to the application of catalytic potentials, the latter resulting in considerably faster but clearly less complete reduction of Cu^+^/Cu^2+^ to Cu^0^. The faster reduction at higher negative potential possibly generated a metallic surface layer, effectively preventing further reduction of subsurface/bulk oxide species.

We find that by specific reduction-oxidation schemes (e.g., potential jump sequence or CV-series), the amount of subsurface/bulk oxygen species can be increased by incorporation of newly formed oxidized material at oxidizing potential that is maintained at negative (catalytic) potentials. The nature of such species is still unresolved, but some characteristics can be deduced. Here, these species are detected by an influence on the Cu K-edge XAS that is in line with Cu oxide species in the first Cu coordination sphere but is not expected for atomic or molecular oxygen species that are not bound to Cu ions. Moreover, since (i) the initially largely oxidized starting Cu foams mainly comprised Cu_2_O or Cu_2_CO_3_(OH)_2_, and (ii) (sub)surface atomic O^0^ or O_2_ species are expected to be thermodynamically unfavorable under reducing conditions (and rather may diffuse to the surface^45^), the trapped oxygen species are most likely Cu^*x*+^-associated O^2−^, OH^−^, and CO_3_^2^^[,71^.

The operando SERS data demonstrated that oxide-to-metal and metal-to-oxide transitions at the Cu foam surface are fast and, in contrast to the bulk processes, can be fully completed within a few seconds (as also illustrated in Fig. 9). During CO_2_RR (at catalytic potentials), we detected the presence of both surface-adsorbed *OH and various C-containing species, which we attribute to adsorbates originating from the electrolyte/buffer system, as confirmed by buffer- and isotope-exchange experiments.

Previous studies have suggested that Cu with oxygen species near the surface can promote CO binding, leading to improved selectivity for C_2+_ products^14,20,36,45,68^. We also found that OD-Cu exhibited a higher Faradaic selectivity towards C_2+_ products in the potential range of −0.6 V to −1.0 V, in comparison to metallic Cu foil. Furthermore, we provide evidence for the coexistence of surface-adsorbed *OH and subsurface/bulk oxygen species in OD-Cu. These oxygen species are believed to play a crucial role in facilitating *CO adsorption, thereby enhancing the selectivity of OD-Cu towards C_2+_ products during CO_2_RR.

## Methods

### Electrodeposition

Electrodeposition of the Cu foams was performed using a modified protocol described previously^35^. In a single compartment cell, two electrodes were utilized without the need for stirring or heating. A Pt mesh served as the counter electrode, while a cleaned and etched Cu foil acted as the working electrode, controlled by a BioLogic SP-240 Potentiostat. Prior to usage, the Cu foils were polished with 0.7 mm diamond paste, cleaned with approximately 10% HNO_3_, and subsequently cleaned in an ultrasonic bath using Milli-Q H_2_O and ethanol. The residual area of the Cu foil is covered by Kapton tape. A negative current of 1.0 A was applied for a defined time, while maintaining a distance of 5 mm between the counter and working electrode. The electrolyte consisted of 0.2 M CuSO_4_ and 1.5 M H_2_SO_4_. Immediately before electrodeposition, the Cu foils were etched with approximately 10% HNO_3_. The resulting electrodeposited foams were washed with Milli-Q water and left to naturally dry in air overnight for future use. Detailed information on the chemicals used, morphology and crystal structure characterizations, and CO_2_RR products analysis is provided in the Supplementary methods.

### Operando XAS experiments

Cu K-edge XAS measurements were performed at beamline KMC-3 of the BESSY-II synchrotron (Helmholtz Center Berlin) with the storage ring operating in top-up mode (300 mA). A standard setup for XAS (I_0/1/2_ ion chambers, double-crystal Si[111] monochromator) was employed and an energy-resolving 13-element silicon-drift detector (RaySpec) placed at 90° to the incident X-ray beam was used for X-ray fluorescence monitoring. XAS spectra to *k* = 14 Å^−1^ were collected within ca. 8 min using a continuous monochromator scan mode. Single-energy X-ray fluorescence data were collected at a time resolution of 1 ms using the SD-detector and in-house data acquisition software. Energy calibration was done using the first inflection point at 8979 eV in the Cu K-edge spectrum of a Cu metal foil. Operando-XAS experiments were performed at 293 K unless otherwise specified, the electrochemical cell was placed in the X-ray beam (shaped to ca. 4×2 mm size by a focusing mirror and slits) and housed a three-electrode arrangement (Working electrode, Cu foam; Counter electrode, Pt mesh; Reference electrode, Ag/AgCl) in 25 mL CO_2_-saturated KHCO_3_ electrolyte (0.1 M, pH 6.8). Further information on the experimental protocols is provided in the SI, section 2.

### Operando SERS experiments

The Cu foams used in the Raman experiments were deposited on Cu foil substrates at 1 A cm^−2^ for 20 s. Raman spectra were collected with a Renishaw inVia Raman spectrometer coupled with a Leica microscope. A laboratory-built electrochemical cell made of polytetrafluoroethylene was interfaced with the Raman microscope for spectro-electrochemical experiments. The cell was equipped with an Ag/AgCl reference electrode and a Pt ring counter electrode and controlled with a SP-200 Potentiostat from BioLogic. A water immersion objective (Leica, 40×, numerical aperture = 0.8) was used to focus and collect the incident and scattered laser light during electrochemical measurements. Dry samples were measured with an objective with 50× magnification. A 633 nm laser with streamline mode (laser focus on a line) was used as the excitation source, with a maximum power of 13 mW. Further experimental details are provided in the SI, section 3.

### Supplementary information

Supplementary information

## Data Availability

All data needed to support our conclusions is presented in the article and the Supplementary Information file.
