# Supplementary material for: Dynamics of bulk and surface oxide evolution in copper foams for electrochemical CO2 reduction
Source: Commun Chem. 2024 Mar 28;7:66. doi: 10.1038/s42004-024-01151-0 (PMC10978924; doi:10.1038/s42004-024-01151-0)
Supplement: Supplementary file 1 — Supplementary information [file 42004_2024_1151_MOESM1_ESM.pdf]

Dynamics of bulk and surface oxide evolution in copper foams for electrochemical CO_2 reduction

Fan Yang[1], Shan Jiang[1], Si Liu[1], Paul Beyer[1], Stefan Mebs[1*], Michael Haumann[1], Christina Roth[2], Holger Dau[1*]

[1] Department of Physics, Freie Universität Berlin, Arnimallee 14, Berlin 14195, Germany

[2] Electrochemical Process Engineering, Universität Bayreuth, Universitätsstraße 30, Bayreuth 95447, Germany

*Corresponding authors: Holger Dau, Stefan Mebs

Table of Contents

1. **Supplementary Methods**
2. **X-ray absorption spectroscopy (XAS) measurements**
3. **Operando surface-enhanced Raman spectroscopy (SERS)**
4. **Supplementary Figures and Tables**
5. **Supplementary Notes**
6. **Supplementary References**

1 Supplementary Methods

1.1 Chemicals

Copper(I) oxide (Cu_2O, >99.99%), copper(II) oxide (CuO), and copper(II) carbonate basic (malachite, $Cu_2CO_3(OH)_2$), copper(II) hydroxide ($Cu(OH)_2$), copper(II) sulfate pentahydrate ($CuSO_4 \cdot 5H_2O$, >99.99%) and potassium bicarbonate ($KHCO_3$, 99%) were purchased from Sigma-Aldrich. Boron nitride (BN, 98%), sulfuric acid (H_2SO_4, 99%) and nitric acid (HNO_3, ACS reagent, 70%) were purchased from Carl Roth. These chemicals were used without further purification. Cu foil substrate (0.1 mm thickness, >99.99% purity; for electrodeposition) and Cu foil (7.5 μm thickness, >99.99% purity; for XAS measurement) were purchased from Goodfellow, graphene sheets (97% carbon) were purchased from Graphene Supermarket. Milli-Q water (18.2 MΩ cm^{-1}) was used to prepare all solutions in this study.

1.2 Morphology characterization

Scanning electron microscopy (SEM) was carried out on a SU8030 scanning electron microscope (Hitachi). Video microscopy was done with a high-resolution camera mounted onto a Raman spectrometer (Renishaw).

1.3 Electrochemical measurements

Electrochemical CO_2 reduction reaction (CO_2RR) experiments for product analysis were performed using a BioLogic SP-240 potentiostat in an H-type two-compartment electrochemical cell, separated by a cation exchange membrane (Nafion 211, 0.125 mm thickness, Alfa Aesar), in CO_2-saturated 0.1 M $KHCO_3$ solution (pH 6.8) under constant CO_2 flow of 20 mL min^{-1}. A mass-flow controller was used to control the flow rate of CO_2. The measured potentials are obtained after resistance compensation and are represented with respect to reversible hydrogen electrode (RHE). The electrolyte in the working electrode compartment was stirred continuously (300 rpm) to achieve maximum mass transport of CO_2 to the electrode surface. A leak-free Ag/AgCl reference electrode was placed close to the working electrode, and a Pt coil was used as the counter electrode. CO_2 gas was also purged to the counter electrode compartment.

Electrode potentials measured on the Ag/AgCl scale ($E_{Ag/AgCl}$) were converted to the RHE scale (E_{RHE}) using the following equation:

$$E_{\mathrm{RHE}} = E_{\mathrm{Ag/AgCl}} + 0.197 + 0.059 * pH \quad \text{(S1)}$$

All the potential values in this study are on RHE scale.

The electrochemical protocol used during the CO_2RR measurements consists of:

1) Purging CO_2 for 10 min to saturate the electrolyte.
2) Resistance measurements by current interrupt (IR) in the BioLogic potentiostat software at open circuit potential (OCP), followed by 4 cyclic voltammetry (CV) sweeps until a stable current was obtained.
3) CO_2 electrolysis measurements were carried out by applying a constant potential for 30 min for gaseous products or 60 min for liquid products.

1.4 Chromatographic analysis of gaseous products

The gaseous products formed in the CO_2RR reaction were identified and quantified using an online gas chromatograph (GC) (Shimadzu GC-2014) coupled with a two-compartment electrochemical cell. The GC was equipped with a thermal conductivity detector (TCD) for the detection of H_2 and other gases. A methanizer in series with a flame ionization detector (FID) was used for detecting CO and

hydrocarbons. Grade 5 argon was used as the carrier gas. The relative faradaic selectivity (FS) of gaseous products was determined by dividing its partial currents by the sum of the partial currents of all detected products.

$$FS_i = \frac{I_i}{\sum I_i} * 100 = \frac{x_i z_i F\dot{n}}{\sum x_i z_i F\dot{n}} * 100 = \frac{x_i z_i}{\sum x_i z_i} * 100 \quad \text{(S2)}$$

where x_i, z_i, F and $\dot{n}$ are the volume fractions of detected products, number of electrons involved for a particular reduction product, Faraday constant and molar flow rate, respectively. The faradaic efficiency (FE, %) of a specific product *i* was calculated as,

$$FE_i = \frac{I_i}{I_t} * 100 = \frac{x_i z_i F\dot{n}}{I_t} * 100 \quad \text{(S3)}$$

where I_t is the total current.

1.5 Quantitative analysis of the liquid products

The quantification of non-volatile liquid CO_2RR products (formate, acetate, etc.) was conducted using an ultra-high-performance liquid chromatograph from Thermos Scientific (Model HPLC+ UltiMate 3000 series) with UV variable wavelength (UltiMate 3000, Dionex) and refraction index (RefractoMax 520, ERC) detectors, and a HyperREZ XP H^+ column, and a mobile phase of 5 mM H_2SO_4(aq). The quantification of volatile liquid CO_2RR products (including methanol, ethanol and propanol) was performed using a gas chromatograph (GC, Thermo Scientific, model Trace 1310) via a heated headspace autosampler together with FID and pulse discharge detectors.

For liquid products,

$$FE_i = \frac{n_i}{n_t} * 100\% \quad \text{(S4)}$$

where n_t is the total number of electrons passed through within 60-min electrolysis. n_i is the number of electrons needed to get x mM of product *i*.

n_i can be calculated by the following equation,

$$n_i = x * N_A * e \quad \text{(S5)}$$

where N_A is the Avogadro constant, e is the number of electrons that are required to form one molecule of product *i* from one CO_2 molecule. x mM product *i* can be calculated from the HPLC results.

1.6 Powder X-ray diffraction (XRD)

XRD measurement was conducted on a D8 Advance instrument by Bruker AXS, using a Bragg Brentano measurement geometry, nickel filtered copper K_α radiation, and a LynxEye Detector. The diffract.eva software by Bruker was used to numerically subtract the $K_{\alpha 2}$ contribution.

2 XAS measurements

Because in XAS experiments, the deposited Cu foams could hardly be distinguished from the underlying Cu metal foil substrate, Cu foams were instead deposited on graphene sheets with 1A cm^{-2} current for 10 s, 20 s, or 30 s. The electrolyte and deposition setup were the same as reported earlier[1] if not specified otherwise. For heat-treatment of Cu foams, the as-prepared Cu foams were heated at 200 ^{o}C for 5 h under aerobic conditions. For copper carbonate hydroxide (CuCarHyd) sample, the as-prepared Cu foam deposited at 1A cm^{-2} for 20 s was immersed in 0.1 M $KHCO_3$ for one week to form malachite-like Cu catalysts. Operando XAS experiments were performed at 293 K in fluorescence mode using back-side illumination. Ex-situ XAS measurements were conducted at 20 K in transmission mode, flattening effect correction was done using the transmission signal.

Collection of XAS reference spectra: Commercial Cu_2O, CuO, and $Cu_2CO_3(OH)_2$ powders were grounded with boron nitride to avoid flattening of spectra in XAS data collection. Specifically, the Cu_2O reference was prepared one day before the measurement, covered by two layers of Kapton tapes, and stored in an air-tight container to prevent the air-oxidation prior to the measurement.

Comment on sensitivity of the reference spectra to air oxidation: Since XAS is a bulk-sensitive method and air oxidation of the bulk material is a very slow process, we consider the XAS reference spectra as being comparably insensitive to air oxidation. For collection of the Raman reference spectra, in the absence of any surface-enhancement effect, the relative contribution of near-surface regions and the material bulk is less clear, because of possible resonance enhancement effects and conceivable reabsorption of Raman light by the colored oxides. The air-oxidation sensitivity of the Raman reference spectra may or may not be more pronounced than for the XAS reference spectra; additional vibrational bands from further oxide species formed by air oxidation of the material cannot be excluded.

Non-air exposed sample used in the ex-situ XAS measurements: The electrodeposited Cu foam was dried using N_2 and then immediately coated with a C-based oil (no relevant X-ray absorption at Cu K-edge) to prevent the air-exposure. Subsequently, the sample was mounted in the sample holder for the measurement.

We approached correction for spectral flattening (or self-absorption) of the fluorescence-detected X-ray absorption spectra. This correction is advisable whenever the total amount of the element of interest in the sample is so high that it results in a major fraction of the exciting X-rays photons being absorbed by the element of interest. The correction relies on simultaneous collection of XAS spectra in fluorescence and absorption (transmission detected) modes on the same sample. The flattening correction was done as follows:

1) Normalization of the fluorescence ($I_F = I_{flu}/I_0$) and absorption ($I_A = -\ln(I_{trans}/I_0)$) spectra in the K-edge region (normalization to unity at ca. 100 eV above the edge-rise onset energy).
2) Division (of data on the same energy axis) of the normalized fluorescence by the measured X-ray absorption (I_F/I_A) provided a 'flattening curve'.
3) Simulation (curve fitting) of the flattening curve using
   $$I_{\mathrm{F}} = I_{\mathrm{max}}[1 - \exp(-\mathrm{k}\, I_{\mathrm{A}})] \quad \text{(S6)}$$
   yielded values for k and I_{max}.
4) Correction of the fluorescence-detected XAS spectra (XANES and EXAFS) at each energy value (E) was done using the k and I_{max} values according to:
   $$I_{\mathrm{F}}^{\mathrm{cor}}(\mathrm{E}) = \ln\left(1 - \frac{I_{\mathrm{F}}(\mathrm{E})}{I_{\mathrm{max}}}\right)/(-\mathrm{k}) \quad \text{(S7)}$$

5) Slight variations of the normalization procedure parameters (background slope subtraction, polynomial order used in normalization) of the absorption spectra was done to achieve best agreement between transmission-detected and flattening-corrected fluorescence-detected XAS spectra.

2.1 Operando XANES experiments

Protocol:

1) Collection of 3-4 XANES spectra at OCP.
2) Acquisition of a series of consecutive XANES spectra at applied potentials.

Collection of a single XANES spectrum took about 227 s.

2.2 Data collection during potential jumps

Electrochemical protocol:

Cu foam deposited on graphene sheet at 1 A cm^{-2} for 20 s was used here.

1) LSV, a fast LSV from OCP/+0.6 V to -0.7 V with 100 mV s^{-1} scan rate.
2) CA, at -0.7 V for 4 min.
3) LSV, a fast LSV from -0.7 V to +0.6 V with 100 mV s^{-1} scan rate.
4) CA, at +0.6 V for 20 min.

The above sequence was repeated 4 times. Potential jump 1 starts from OCP to -0.7 V. Jumps 2-4 start from +0.6 V to -0.7 V. Importantly, in preliminary experiments, we observed that an abrupt potential switch between -0.7 V and +0.6 V results in significant sample degradation. Consequently, we opted for a gradual change by a linear sweep of the potential with 100 mV s^{-1}. It is crucial to determine an optimal scan rate range: if it is too slow, it may obscure the Cu redox kinetics induced by the potential change; if it is too fast, it can lead to detachment of parts of the Cu foam material. A scan rate of 100 mV s^{-1} is an appropriate parameter that aligns well with the goals of the experiment, that is, detection of the Cu redox-state changes after the potential change without the destructive effects of a sudden potential change.

X-ray fluorescence counts at an incident energy at the Cu K-edge (9013 eV) were collected continuously during the potential jump protocol. The original time resolution of XAS data collection was 1 ms per data point, while the time-resolution presented in the corresponding figures is 100 ms for clarity (averaging over 100 data points).

2.3 Data collection and processing during CV cycles

Cu foam deposited on graphene sheet at 1 A cm^{-2} for 20 s was used here. The X-ray fluorescence intensities at four distinct excitation energies (8960, 8982, 9013, 9020 eV) were recorded by means of rapid monochromator movements between the four energies. One set of the corresponding four X-ray fluorescence intensities was recorded every 9 s. The data collection at four excitation energies was continuously carried out during 2 CV cycles (starting at OCP of about +0.6 V, followed by a forward scan down to the potential limitation of -0.7 V, and a backward scan up to the potential limitation of +0.8 V, 1 mV s^{-1}).

The normalized X-ray fluorescence intensity at 8982 eV and 9013 eV was calculated as follows:

$$I_{\text{norm,energy}} = \frac{I_{\text{raw,energy}} - I_{\text{raw,8960}}}{I_{\text{raw,9020}} - I_{\text{raw,8960}}} \quad \text{(S8)}$$

Where energy is either 8982 eV or 9013 eV; $I_{\text{raw, energy}}$ is the detected raw fluorescence intensity at the selected energy.

$I_{\text{raw, 9020}}$ and $I_{\text{raw, 8960}}$ are the detected raw (not normalized) fluorescence intensities at 9020 eV and 8960 eV, respectively.

In **Fig. 5**, the negative current within the Cu_2O reduction region is integrated to determine the charge for reduction, while the positive current within the Cu oxidation region is integrated to determine the charge for oxidation. The integration ranges were chosen such that outside the integration range the X-ray signal was constant, which indicates the absence of redox-state changes of the Cu material.

3 Operando SERS

Cu foam deposited on Cu foil at 1 A cm^{-2} for 20 s was used for operando SERS experiments. For Raman spectra collected at -0.2 V and +0.6 V (**Fig. 6** and Supplementary Figure 22), a 633 nm laser with 50% of maximal power of ca. 13 mW was used, the data collection time was 1 s for each spectrum (136-2267 cm^{-1} range). For the other Raman experiments, a 633 nm laser with 10% of maximal power of ca. 13 mW was used. Two ranges of spectra were acquired separately, 106-1967 cm^{-1} and 1926-3384 cm^{-1}, and after data analysis presented as a single spectrum. Five accumulations were averaged per spectrum; the data collection time was 5 s for each accumulation.

Collection of Raman reference spectra: commercial Cu_2O, CuO, $Cu(OH)_2$ and $Cu_2CO_3(OH)_2$ powders were used without any further treatment. Notably, the Cu_2O reference spectrum was collected immediately after extracting the Cu_2O powder from the air-tight tube for preventing the air-oxidation.

To determine the peak intensity of Raman bands, the following steps were performed:

1) The maximum value within the selected peak region is identified.
2) A data point before and a data point after the maximum value are selected.
3) These three points represent the actual components of the selected peak, excluding any artifacts or oscillations.
4) The peak intensity is obtained by averaging the values of these three points.

4 Supplementary Figures and Tables

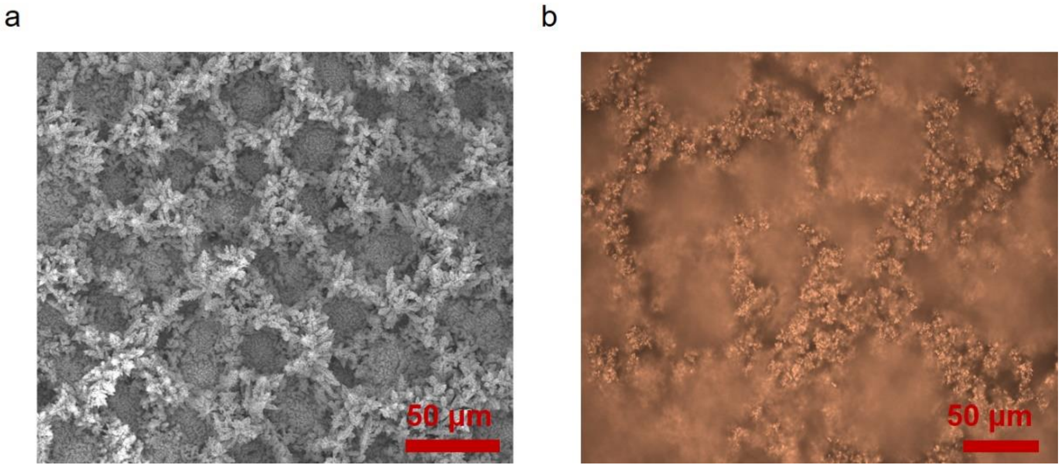

Supplementary Figure 1. Morphology of Cu foam deposited on Cu foil at 1 A cm^{-2} for 20 s. a) SEM and b) video microscopic images.

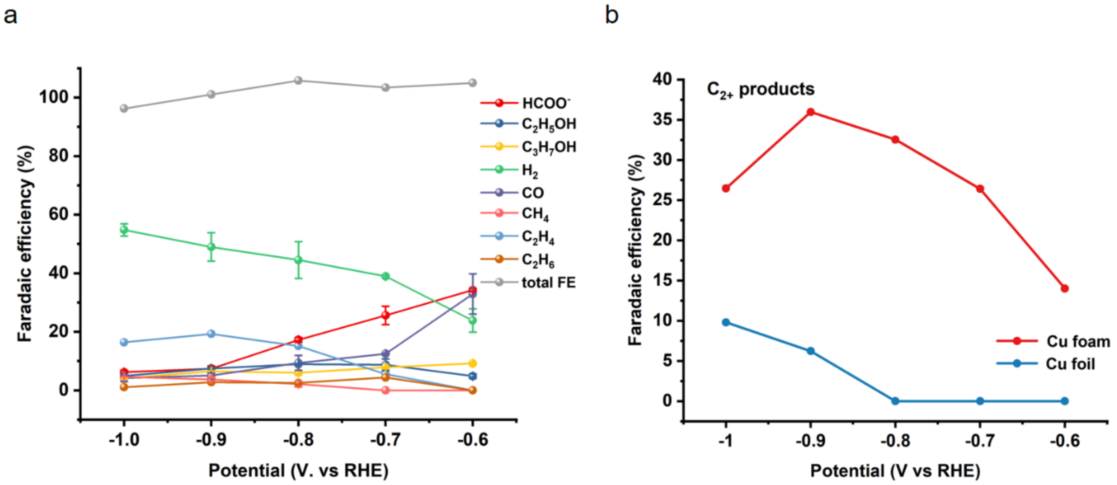

Supplementary Figure 2. CO_2RR performance of Cu foam in CO_2-saturated 0.1 M $KHCO_3$ (pH 6.8). Cu foam was deposited on Cu foil at 1 A cm^{-2} for 20 s. a) Faradaic selectivity of all gaseous and liquid products (CO, CH_4, C_2H_4, C_2H_6, $HCOO^-$, C_2H_5OH, C_3H_7OH) of CO_2 electroreduction along with H_2 as a function of applied potential for Cu foam. Gaseous products were analyzed after 30 min electrolysis. Liquid products were analyzed after 1 h electrolysis. d) Faradaic efficiency for C_{2+} products of Cu foam (red) and Cu foil (blue) at different applied potentials. When error bars are presented, data are represented as mean ± standard deviation of at least three individual experiments.

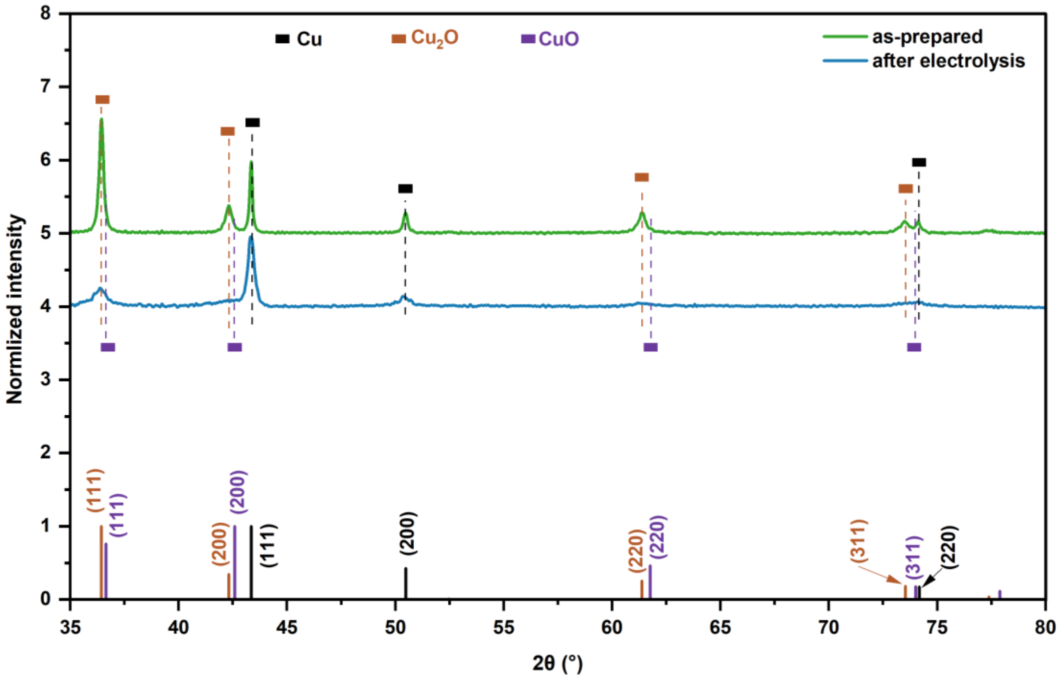

Supplementary Figure 3. Representative XRD patterns of Cu foam (1A cm^{-2} for 20 s) in as-prepared state (green line) and after-electrolysis state (blue line; after 1 h reaction at -0.7 V). Main reflections are indicated; reference patterns from International Centre for Diffraction Data; Cu: PDF 03-065-09026; Cu_2O: PDF 01-071-3645; CuO: PDF 01-078-0428. The data are normalized by Cu(111).

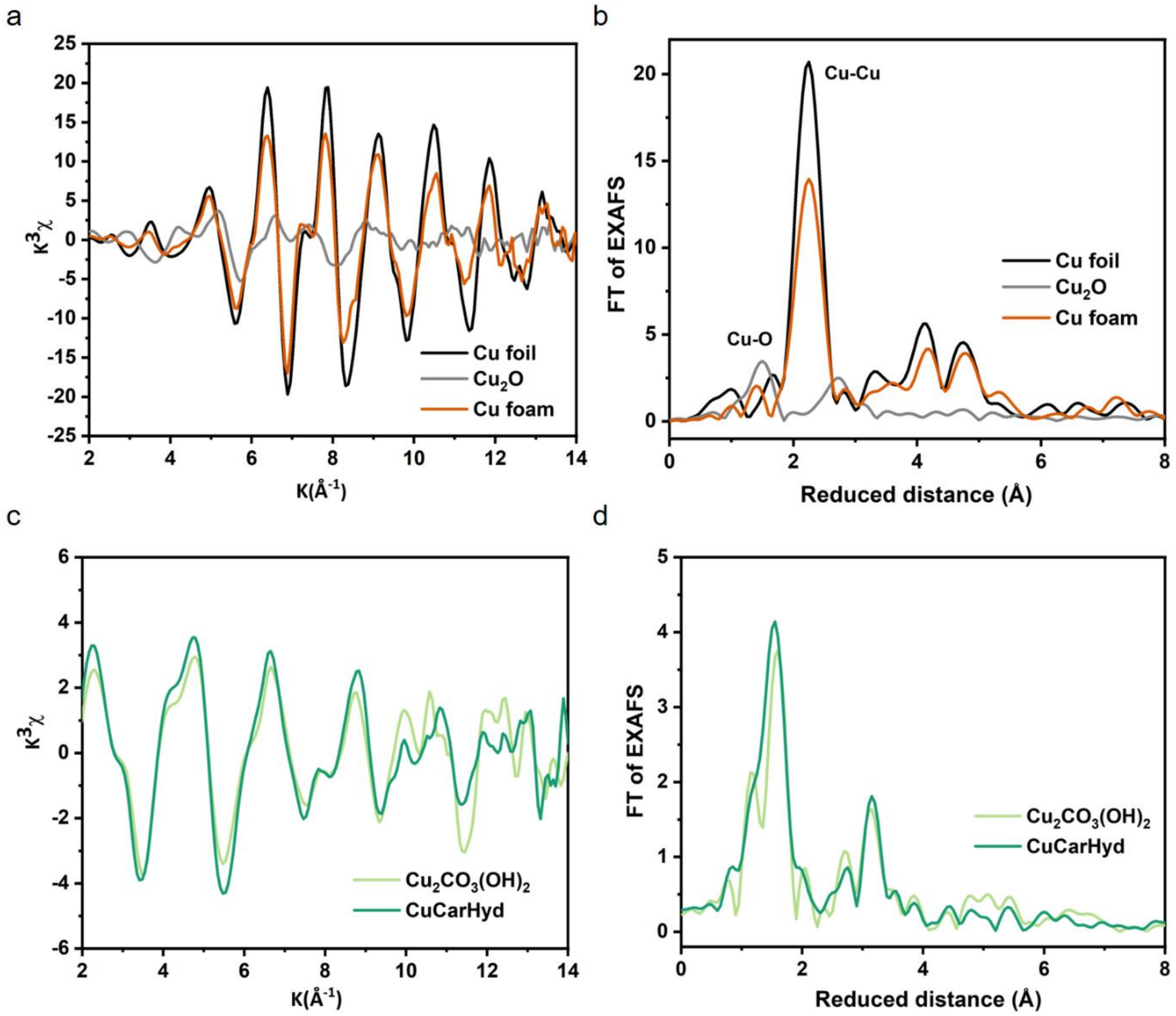

Supplementary Figure 4. Cu K-edge EXAFS of as-prepared Cu foam and CuCarHyd. a) k-space and b) FT of EXAFS for Cu foam (orange) deposited on graphene sheet at 1 A cm^{-2} for 20 s, Cu_2O powder (grey) and Cu foil (black). c) k-space and d) FT of EXAFS for CuCarHyd (dark green) and $Cu_2CO_3(OH)_2$ (light green).

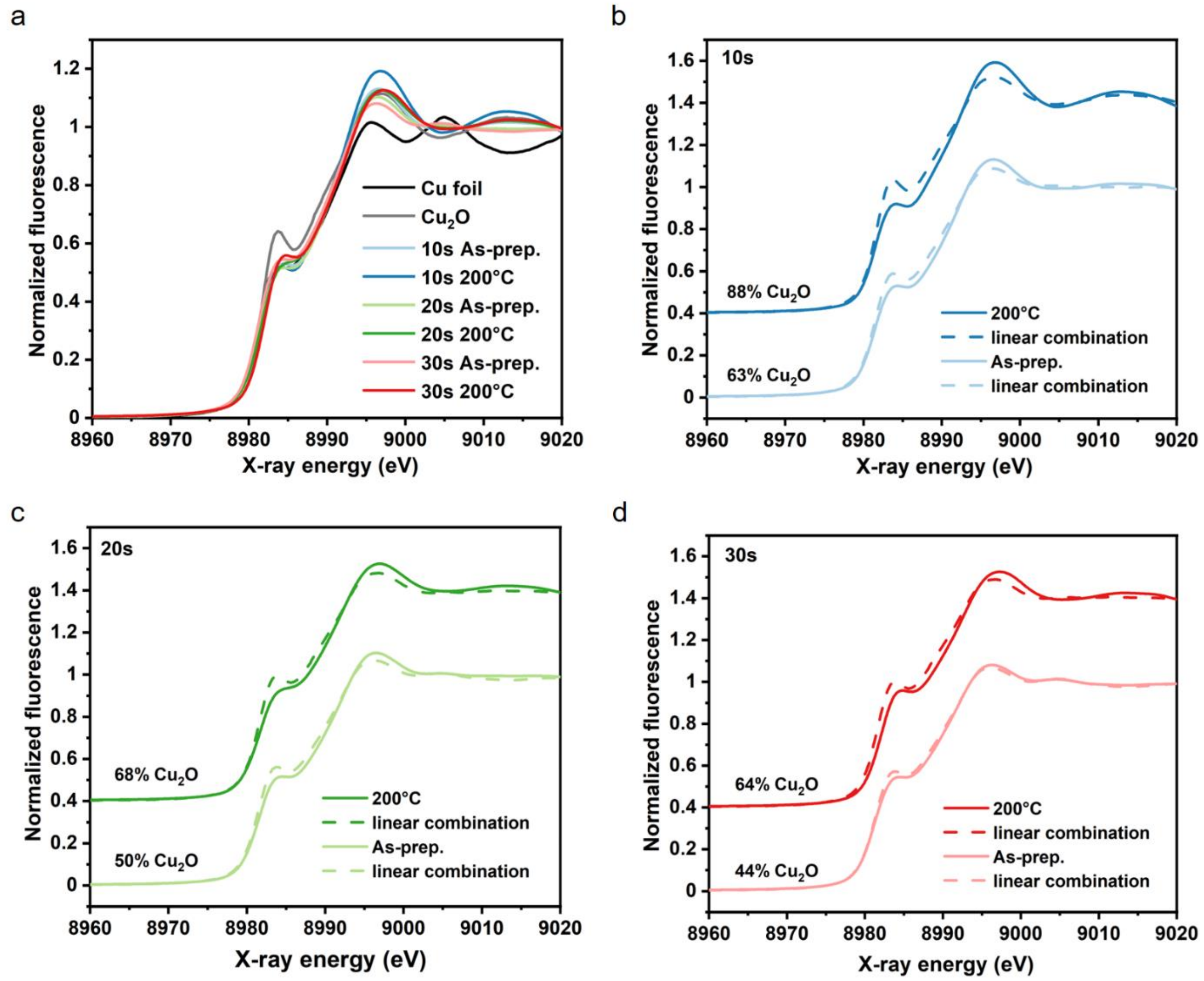

Supplementary Figure 5. Cu K-edge XANES spectra of Cu foams with 1 A cm^{-2} deposition current. As-prep: as prepared Cu foam deposited on graphene sheets for 10 s, 20 s, or 30 s; 200°C: the as-prepared Cu foams were heat-treated at 200°C for 5 h. Since Cu foam looks neither like Cu foil or Cu_2O, nor like a combination of both, the completely reduced Cu foam with 10 s deposition time (potential of -0.2 V applied until non-changing spectra were achieved) was considered to be the best reference for linear combination (denoted Cu^0 in the following). a) XANES spectra of as-prepared Cu foams and 200ºC-treated Cu foams. Cu foil (black) and Cu_2O (grey) spectra are shown for comparison. b) Spectra of as-prepared Cu foams (light blue, solid line), 200°C-treated Cu foams (dark blue, solid line) with 10 s deposition time, and linear combination of 63% Cu_2O and 37% Cu^0 spectra (light blue, dashed line) or 88% Cu_2O and 12% Cu^0 spectra (dark blue, dashed line). c) Spectra of as-prepared Cu foams (light green, solid line), 200°C-treated Cu foams (dark green, solid line) with 20 s deposition time, and linear combination of 50% Cu_2O and 50% Cu^0 spectra (light green, dashed line) or 68% Cu_2O and 32% Cu^0 spectra (dark green, dashed line). d) Spectra of as-prepared Cu foams (light red, solid line), 200°C-treated Cu foams (labeled as 200°C, dark red, solid line) with 30 s deposition time, and linear combination of 44% Cu_2O and 56% Cu^0 spectra (light red, dashed line) or 64% Cu_2O and 36% Cu^0 spectra (dark red, dashed line). The spectra in b), c) and d) are shifted vertically for visual clarity.

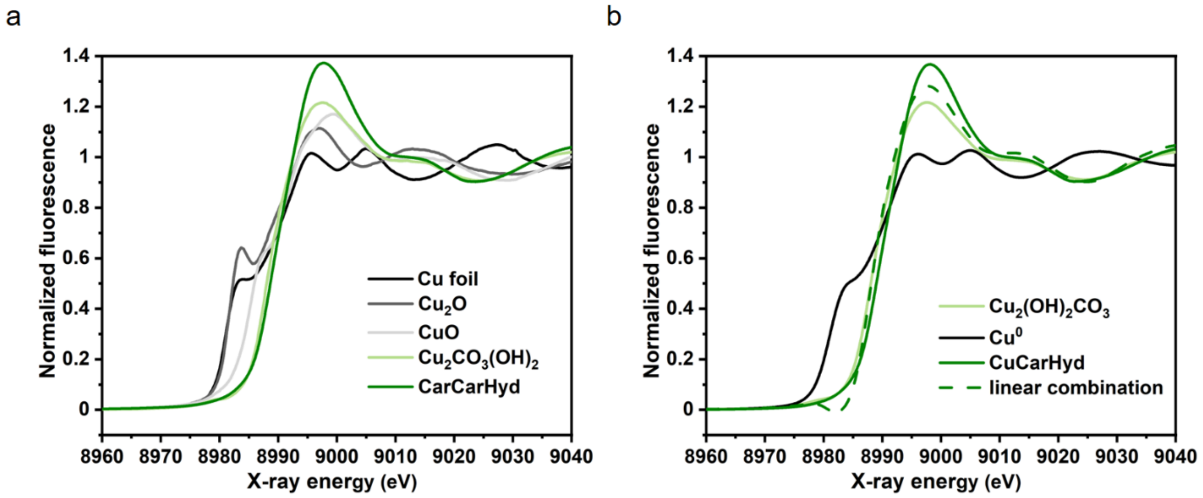

Supplementary Figure 6. Cu K-edge XANES of CuCarHyd and reference spectra collected at OCP in CO_2-saturated 0.1 M $KHCO_3$ solution (pH 6.8). a) Spectra of CuCarHyd sample (dark green), Cu foil (black), Cu_2O (grey), CuO (light grey) and $Cu_2CO_3(OH)_2$ (light green). b) Spectra of CuCarHyd sample (dark green, solid line), $Cu_2CO_3(OH)_2$ (light green), Cu^0 (black), and 100% $Cu_2CO_3(OH)_2$ spectrum (green, dashed line). Cu^0: spectrum of a completely reduced CuCarHyd sample.

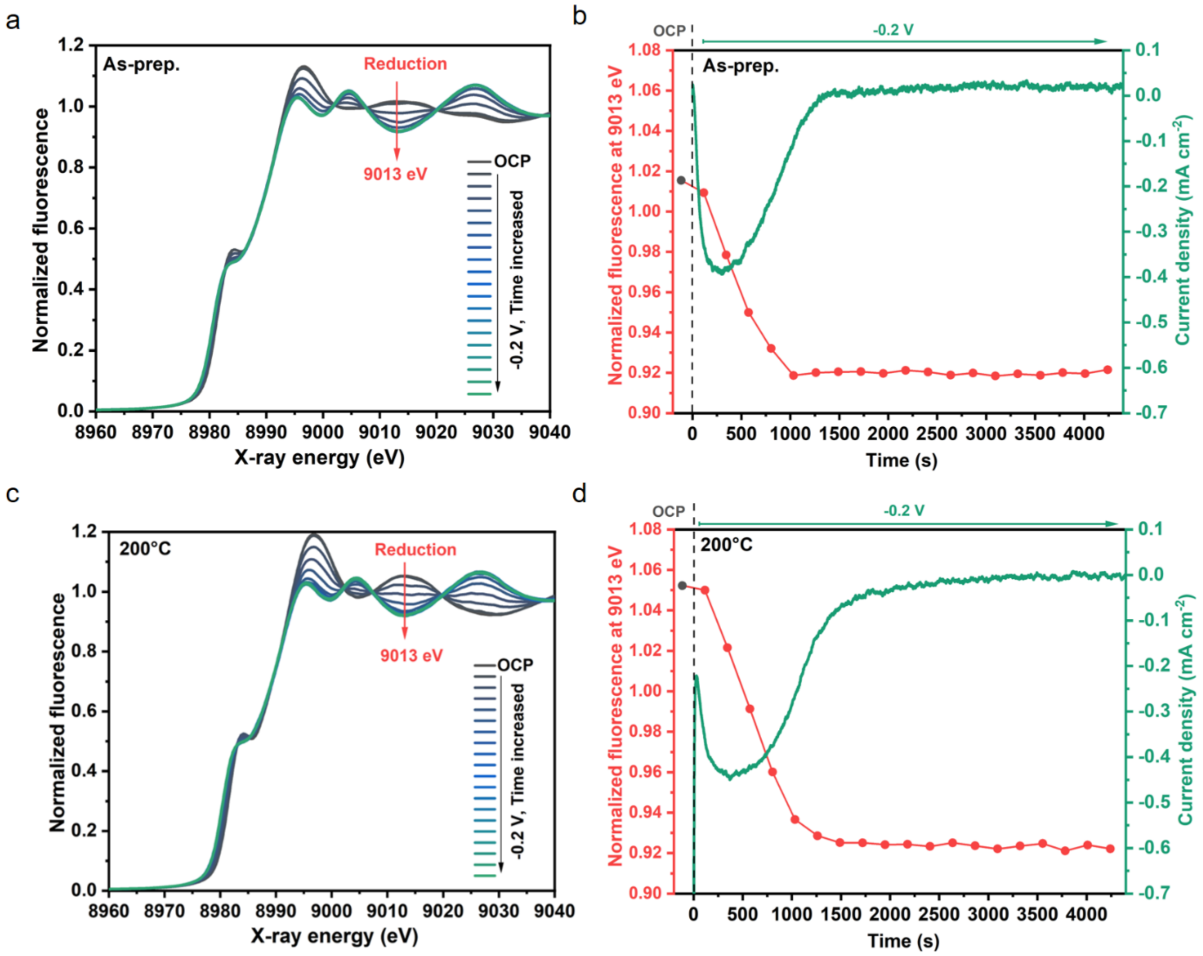

Supplementary Figure 7. Reduction of Cu foam at -0.2 V in CO_2-saturated 0.1 M $KHCO_3$ solution (pH 6.8). Cu foams were deposited on graphene sheets at 1 A cm^{-2} for 10 s. Time-series of Cu K-edge XANES spectra for a) as-prepared Cu foam and c) 200°C-treated Cu foam. The time interval between

spectra is 227 s. Time courses of normalized fluorescence intensity at 9013 eV and current density for b) as-prepared Cu foam and d) 200°C-treated Cu foam. The time spacing of data points in b) and d) is 227 s.

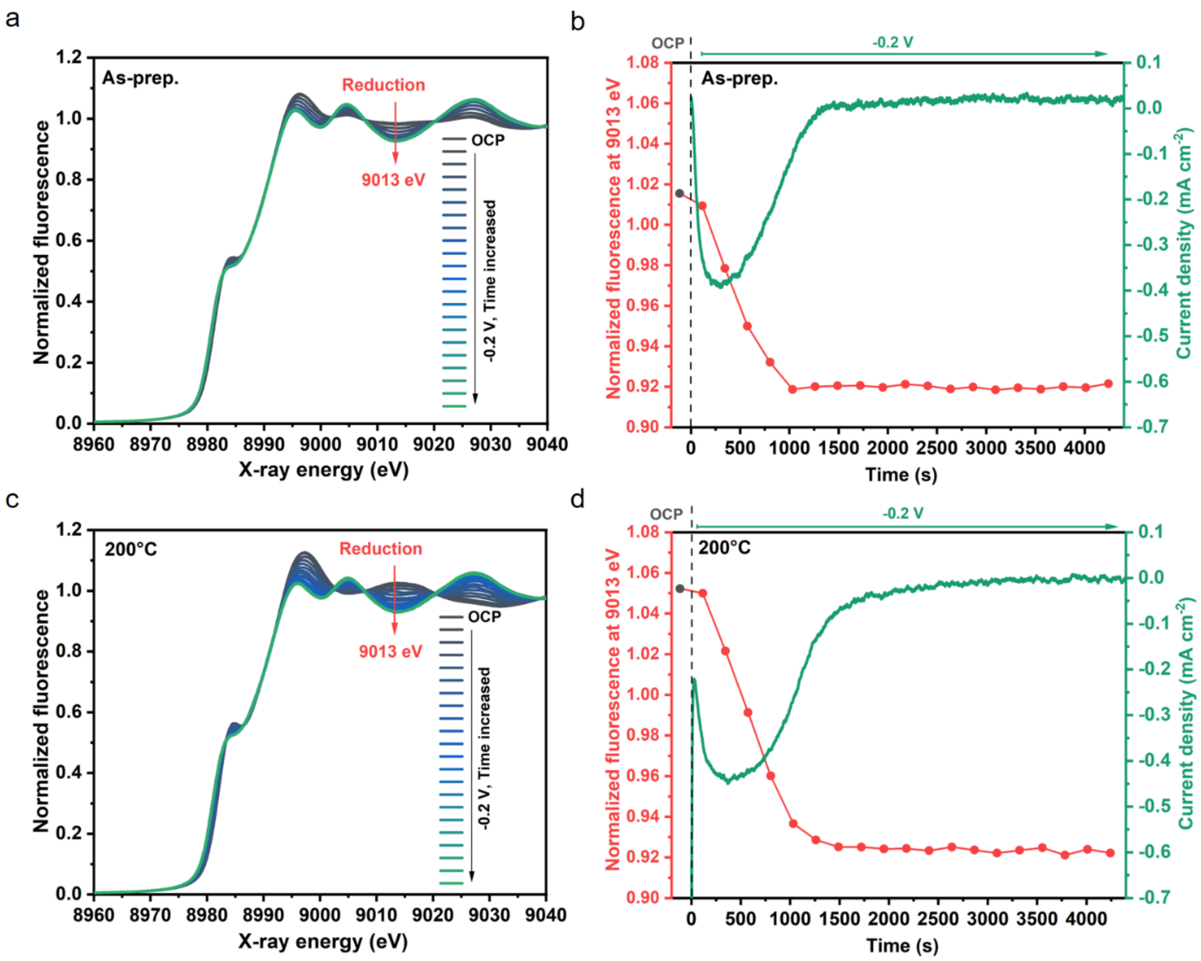

Supplementary Figure 8. Reduction of Cu foam at -0.2 V in CO_2-saturated 0.1 M $KHCO_3$ solution (pH 6.8). Cu foams were deposited on graphene sheets at 1 A cm^{-2} for 30 s. Time-series of Cu K-edge XANES spectra for a) as-prepared Cu foam and c) 200ºC-treated Cu foam. The time interval between spectra is 227 s. Time courses of normalized fluorescence intensity at 9013 eV and current density for b) as-prepared Cu foam and d) 200ºC-treated Cu foam. The time spacing of data points in b) and d) is 227 s.

Supplementary Table 1. Properties of Cu foams deposited on graphene sheets at 1 A cm^{-2} and of CuCarHyd.

Sample		Cu^0 (%)	Cu_2O or $Cu_2CO_3(OH)_2$ (%)	Half-time (s)
Cu foam 10 s	As-prep.	37	63	440
	200°C	12	88	600
Cu foam 20 s	As-prep.	50	50	490
	200°C	32	68	650
Cu foam 30 s	As-prep.	56	44	590
	200°C	36	64	1190
CuCarHyd		----	100	1940

The contents of Cu^0 and $Cu_2O/Cu_2CO_3(OH)_2$ were obtained from XANES analysis. Cu foam spectra can be modeled using Cu^0 and Cu_2O spectra. The CuCarHyd spectrum can be modeled using Cu^0 and $Cu_2CO_3(OH)_2$ spectra. The initial intensity value was determined by averaging 3-4 spectra collected at OCP, resulting in the mean spectral intensity at 9013 eV (Cu foam)/9025 eV (CuCarHyd). Similarly, the final intensity value was obtained by averaging the last 4 spectra collected at -0.2 V, resulting in the mean spectral intensity at 9013 eV (Cu foam)/9025 eV (CuCarHyd). The maximum value represents the difference between the averaged initial intensity and final intensity. The half-time refers to the duration it takes for the fluorescence intensity to decrease or increase by half.

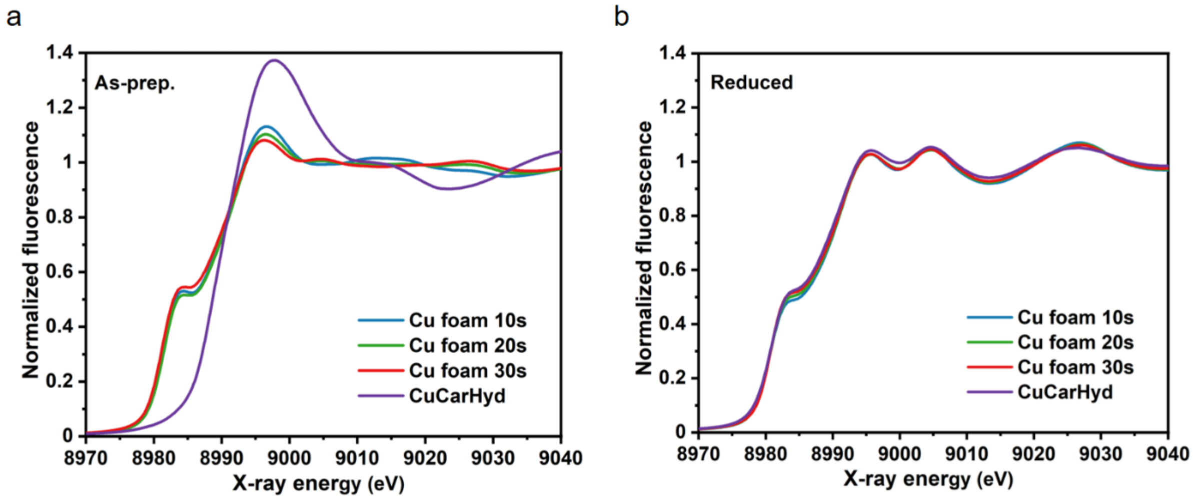

Supplementary Figure 9. Cu K-edge XANES of Cu foams and CuCarHyd collected in CO_2-saturated 0.1 M $KHCO_3$ solution (pH 6.8). Cu foams were deposited on graphene sheets at 1 A cm^{-2} for 10 s, 20 s or 30 s. a) XANES of Cu foams and CuCarHyd collected at OCP condition. b) Spectra of Cu foams and CuCarHyd collected at -0.2 V (the potential was applied until non-changing spectra were reached).

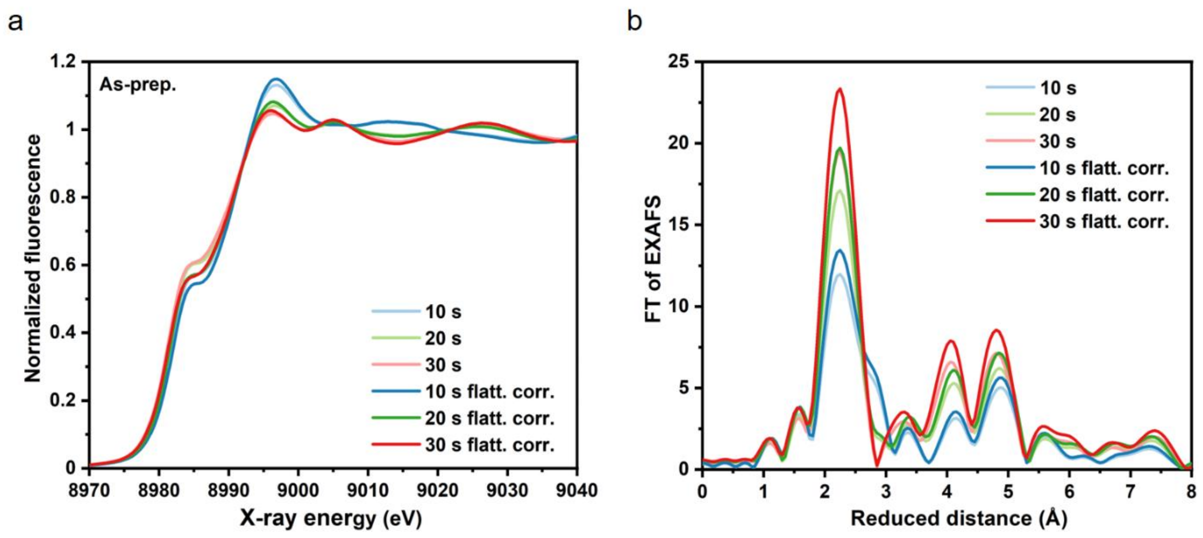

Supplementary Figure 10. Ex-situ Cu K-edge XAS of Cu foams collected at 20 K. Cu foams were deposited on graphene sheets at 1 A cm^{-2} for 10 s, 20 s and 30 s. a) XANES and b) EXAFS spectra of Cu foams with and without flattening correction (flatt. corr.).

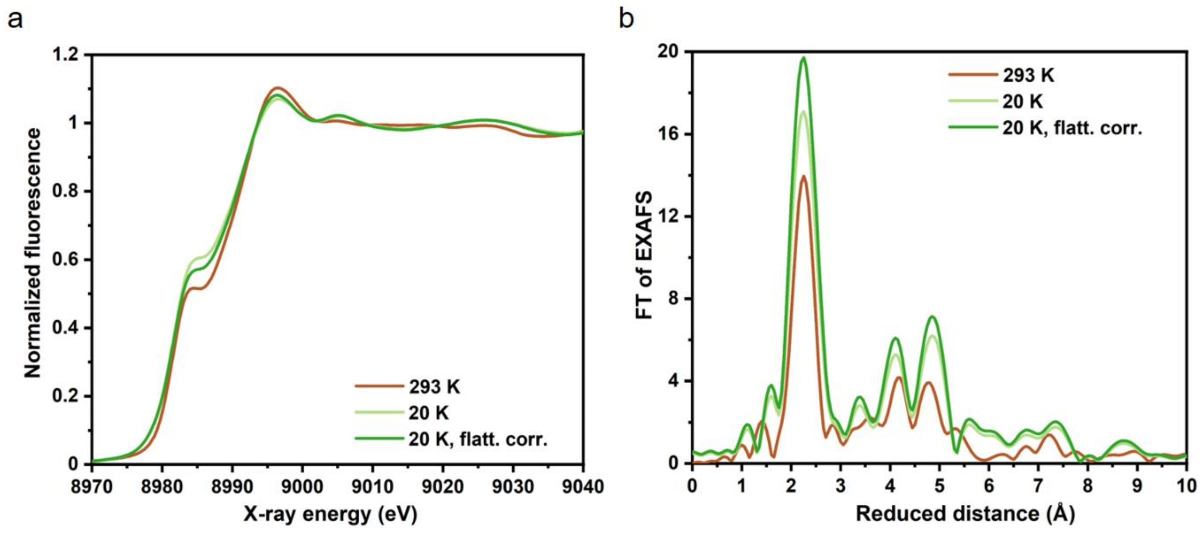

Supplementary Figure 11. Cu K-edge a) XANES and b) EXAFS spectra of as-prepared Cu foam. Cu foam was deposited on graphene sheets at 1 A cm^{-2} for 20 s. The 293 K spectrum (taken from Supplementary Figure 9a) was collected under operando conditions, preventing the acquisition of transmission signals required for flattening correction.

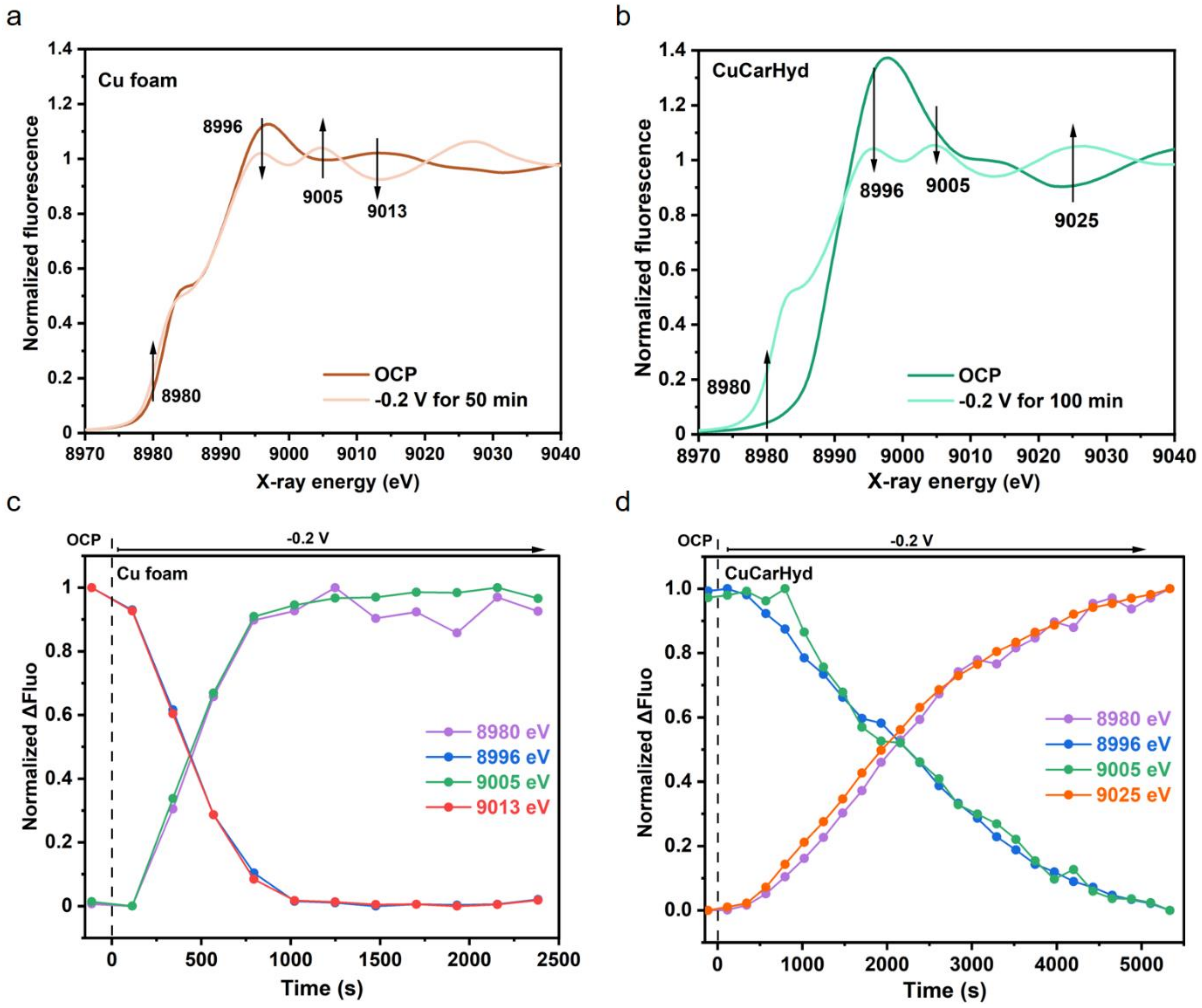

Supplementary Figure 12. Comparison of Cu foam and CuCarHyd. Cu foam was deposited on graphene sheet at 1 A cm^{-2} for 20 s, followed by heat-treatment at 200ºC for 5 h. Cu K-edge XANES of a) Cu foam and b) CuCarHyd collected at OCP condition or at -0.2 V in CO_2-saturated 0.1 M $KHCO_3$ solution (pH 6.8). Time courses of normalized fluorescence changes (ΔFluo) at selected energies are shown for Cu foam in c) and for CuCarHyd at -0.2 V in d). The time spacing of data points in c) and d) is 227 s.

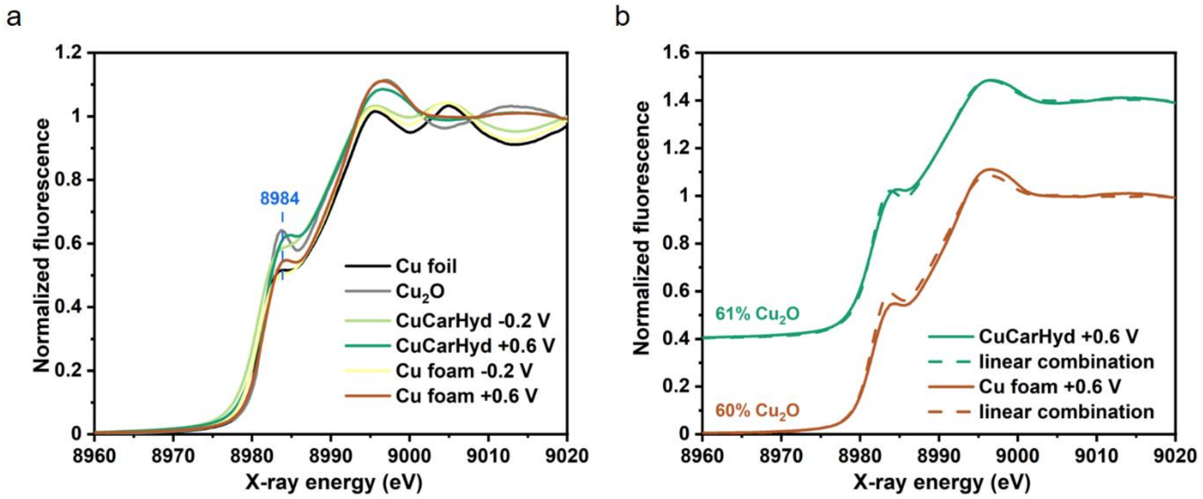

Supplementary Figure 13. Cu K-edge XANES of Cu foams and CuCarHyd collected in CO_2-saturated 0.1 M $KHCO_3$ (pH 6.8). Cu foam was deposited on graphene sheet at 1 A cm^{-2} for 20 s. a) XANES of Cu foam and CuCarHyd collected at -0.2 V or +0.6 V (potentials were applied until non-changing spectra were reached), spectra of Cu foil and Cu_2O are shown for comparison. Pre-edge features at 8984 eV are marked by a dashed line. b) Spectra of Cu foam at +0.6 V (brown, solid line), CuCarHyd at +0.6 V (green, solid line), and linear combination of 60% Cu_2O and 40% Cu^0 (brown, dashed line) or 61% Cu_2O and 39% Cu^0 (green, dashed line).

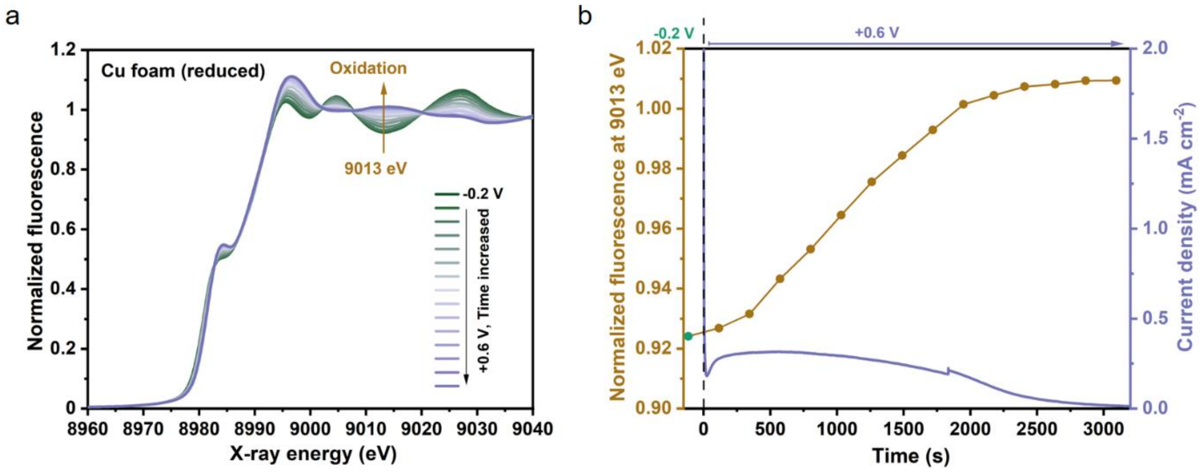

Supplementary Figure 14. Oxidation of reduced Cu foam at +0.6 V in CO_2-saturated 0.1 M $KHCO_3$ (pH 6.8). Cu foam was deposited on graphene sheet at 1 A cm^{-2} for 20 s. Cu foam was oxidized at +0.6 V until non-changing spectra were reached. a) Time series of Cu K-edge XANES spectra and b) time courses of normalized fluorescence intensity at 9013 eV and current density for Cu foam. The time interval between the spectra in a) is 227 s. The half-time for the reoxidation in b) is 1030 s.

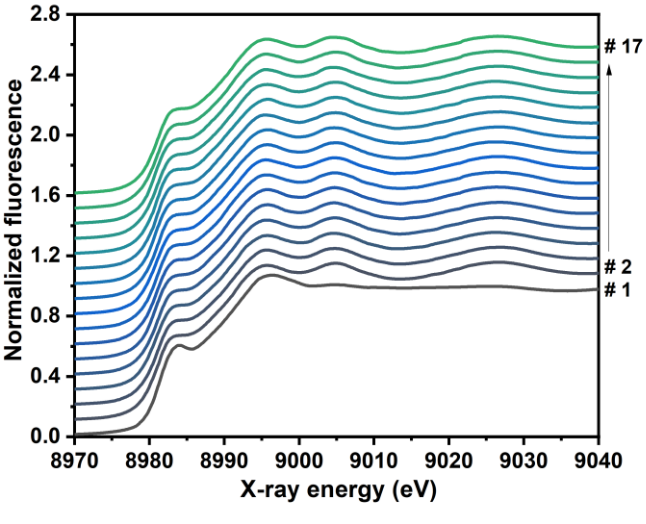

Supplementary Figure 15. Replot of **Fig. 4a** with vertical offset for visual clarity. Time series of Cu K-edge XANES spectra for Cu foam at -0.7 V in CO_2-saturated 0.1 M $KHCO_3$, pH 6.8. The time interval between the spectra is 227 s.

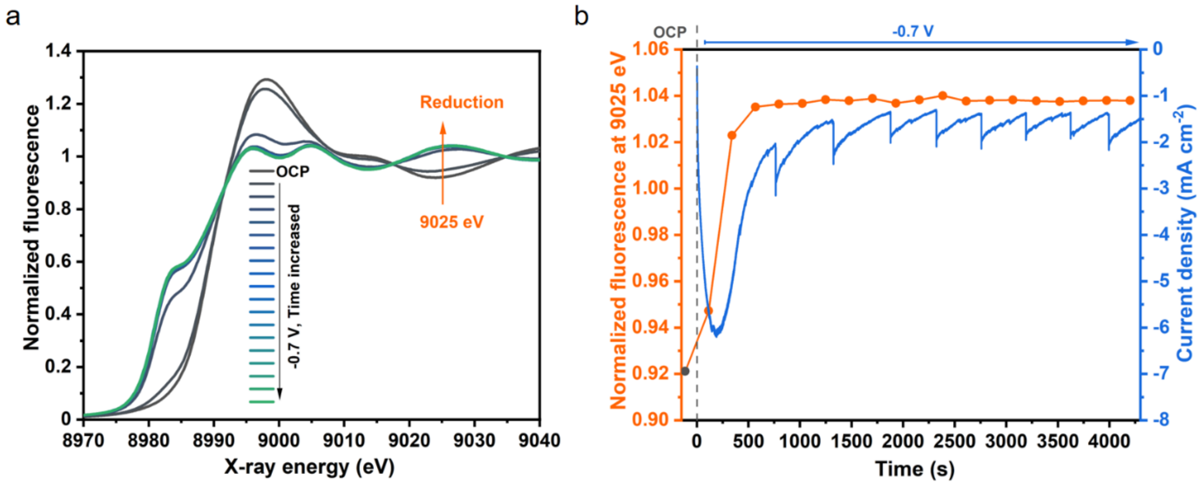

Supplementary Figure 16. Reduction of CuCarHyd at -0.7 V in CO_2-saturated 0.1 M $KHCO_3$ solution (pH 6.8). a) Time series of Cu K-edge XANES spectra, the time interval between spectra is 227 s. b) Time courses of normalized fluorescence intensity at 9025 eV and current density.

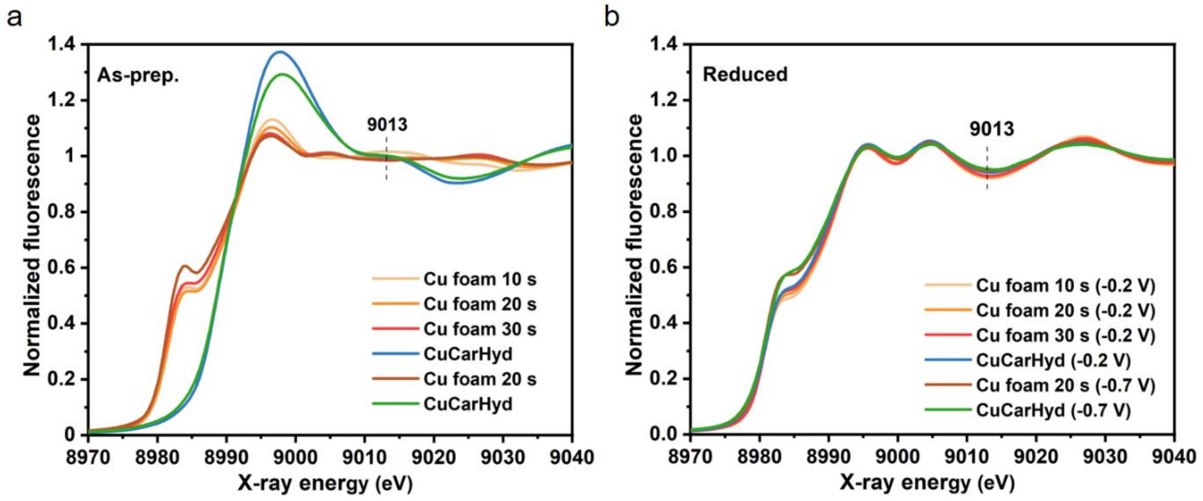

Supplementary Figure 17. Cu K-edge XANES of Cu foams and CuCarHyd collected in CO_2-saturated 0.1 M $KHCO_3$ solution (pH 6.8). Cu foams were deposited on graphene sheets at 1 A cm^{-2} for 10 s, 20 s or 30 s. XANES of as-prepared Cu foams and CuCarHyd collected at a) OCP condition. b) Spectra of Cu foams and CuCarHyd collected at -0.2 V and -0.7 V (the potential was applied until non-changing spectra were reached). The same color used in a) and b) represents the same sample.

Supplementary Table 2. Fluorescence intensities at 9013 eV for Cu foams and CuCarHyd at different potentials

	OCP	-0.2 V	-0.7 V
Cu foam 10 s	1.016	0.920	
Cu foam 20 s	0.994; 0.987	0.924	0.947
Cu foam 30 s	0.984	0.928	
CuCarHyd	0.998; 1.002	0.941	0.953

The fluorescence intensities at 9013 eV are obtained from Supplementary Figure 17. The numbers presented in the same color correspond to identical samples at different potentials.

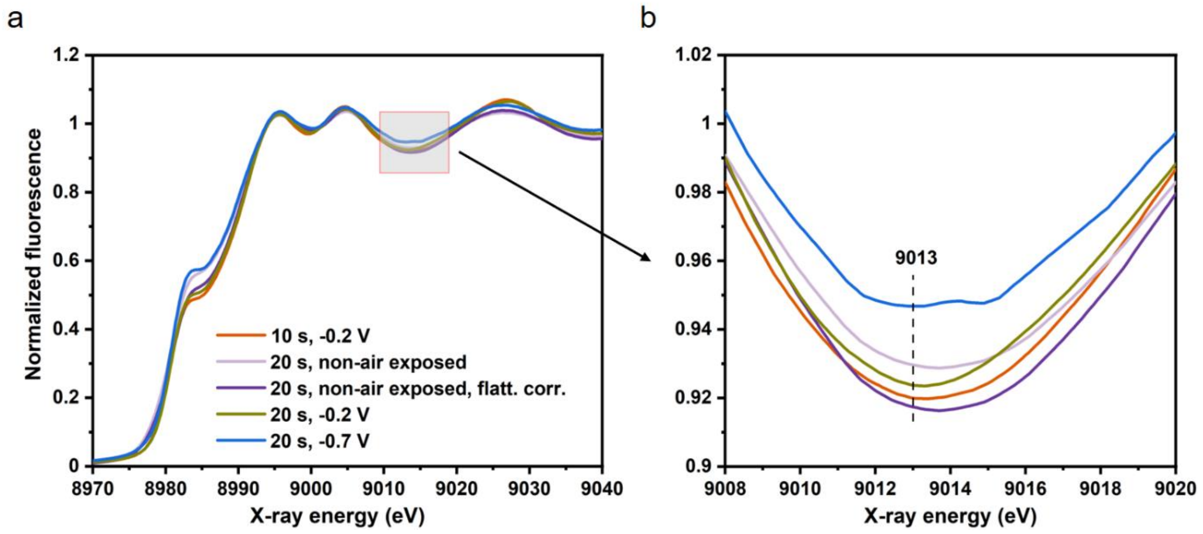

Supplementary Figure 18. Cu K-edge XANES spectra of Cu foams are displayed in a) with an enlarged view in b). Cu foams were deposited on graphene sheets at 1 A cm^{-2} for 10 s or 20 s. Non-air exposed sample was measured at 20 K. Spectra of Cu foams were collected at -0.2 V or -0.7 V (the potential was applied until non-changing spectra were reached) in CO_2-saturated 0.1 M $KHCO_3$ solution (pH 6.8) at 293 K.

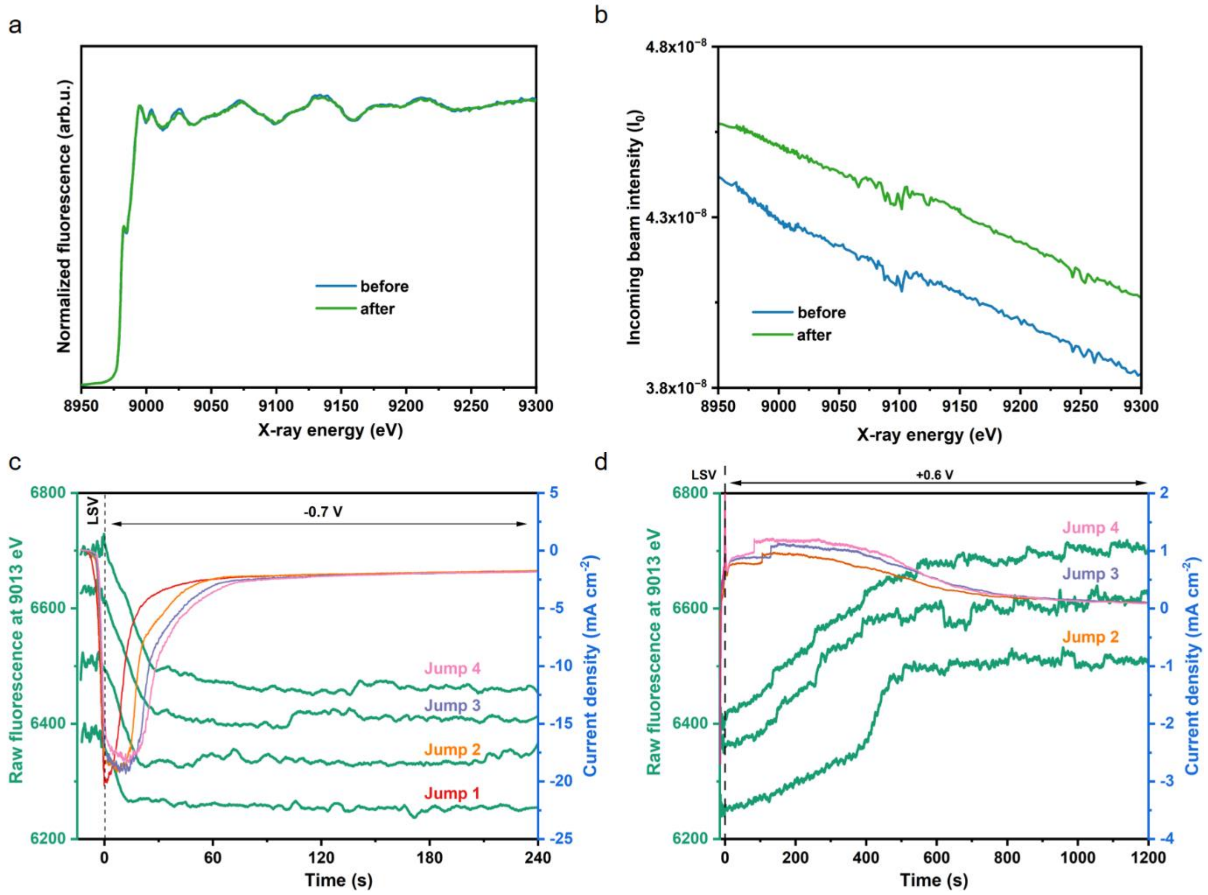

Supplementary Figure 19. In-situ detection of X-ray fluorescence changes at the Cu K-edge for Cu foam during CAs in CO_2-saturated 0.1M $KHCO_3$ (pH 6.8). Cu foam was deposited on graphene sheet at 1 A cm^{-2} for 20 s. a) Cu K-edge XAS spectra of Cu foam and b) respective incoming beam intensity (I_0) before and after the potential steps, spectra were collected in an EXAFS scan, the X-ray fluorescence

signal in a) was normalized by I_0. Before: the as-prepared Cu foam was measured at OCP before electrochemical application. After: after the potential jumps, the potential was back to OCP, Cu foam was measured at OCP. c) Time-courses of X-ray fluorescence intensity at 9013 eV (green thicker lines) and current density (thinner lines) at -0.7 V for 240 s. d) Time-courses of X-ray fluorescence intensity at 9013 eV (green thicker lines) and current density (thinner lines) at +0.6 V for 1200 s. c) and d) Data are taken from **Fig. 4c**. Potential jumps from oxidative potentials (OCP or + 0.6 V) to reductive potential (-0.7 V) were conducted four times; jump 1 was from OCP to – 0.7 V, jumps 2-4 were from + 0.6 V to -0.7 V. Current during Jump 1: red line; jump 2: orange line; jump 3: purple line; jump 4: pink line; a fast LSV (100 mV s^{-1} scan rate, 13 s from -0.7 V to 0.6 V) was applied in between the jumps to avoid sudden changes in voltage from destabilizing the catalyst; the raw X-ray fluorescence signal was not normalized by I_0.

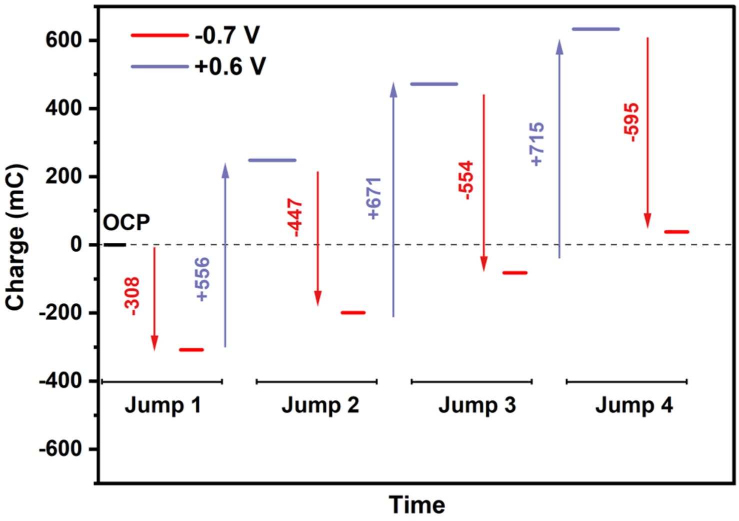

Supplementary Figure 20. Integrated charges during the potential jumps, current vs time are taken from Supplementary Figure 19c-d**.** For reduction operated at -0.7 V, the current collected in the last 40 seconds was averaged to obtain the catalytic current I_s. The measured current at real-time was then subtracted by I_s to obtain I_r. The integrated charge (red) represents the integration of the I_r values over time during the 60-second reaction at -0.7 V. For oxidation operated at +0.6 V, the integrated charge (purple) represents the integration of the real-time current (without any background subtraction) over time during the 1200-second reaction at +0.6 V.

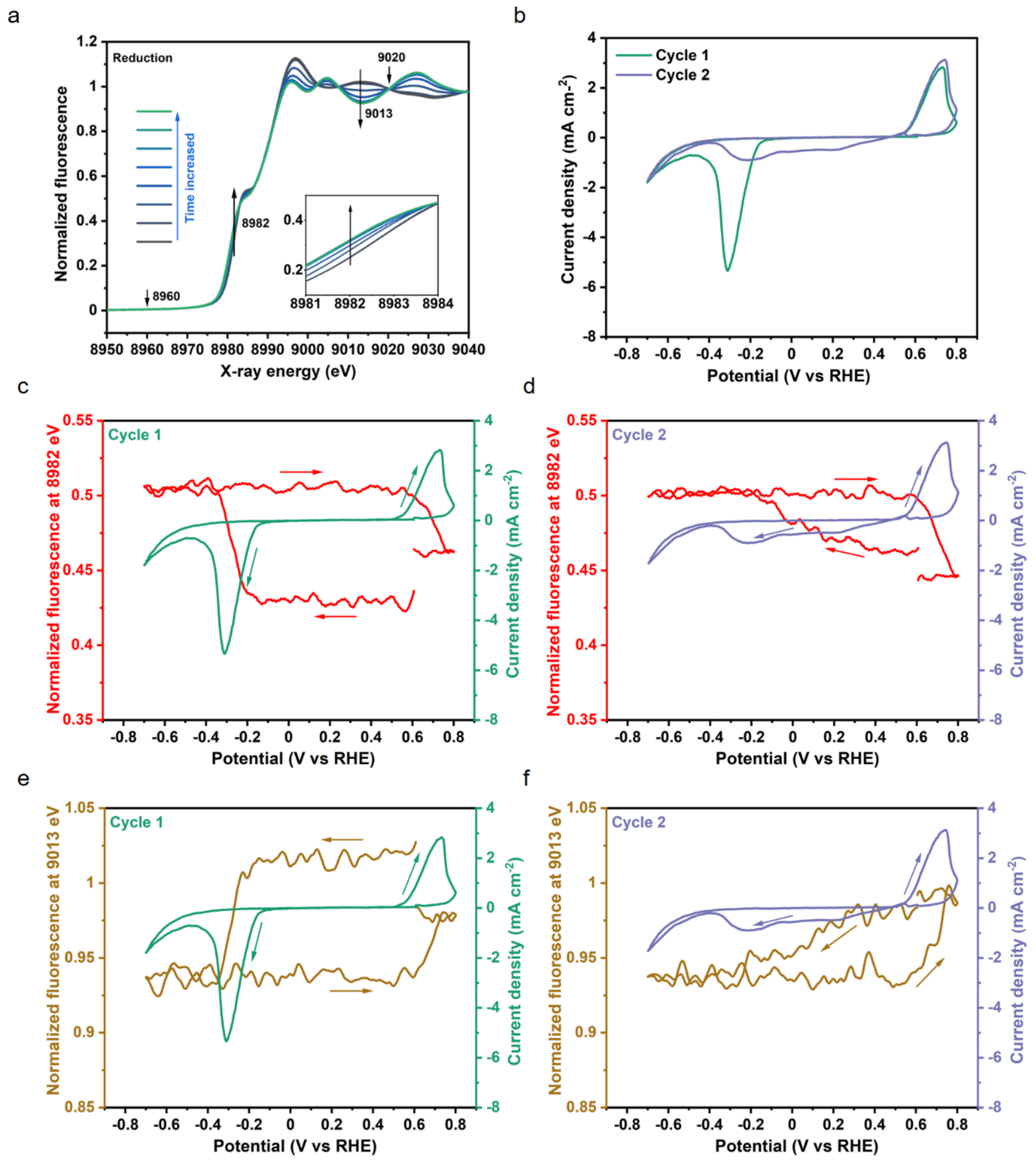

Supplementary Figure 21. In-situ XAS on Cu foam during 2 CV cycles (1 mV s^{-1}) in CO_2-saturated 0.1 M $KHCO_3$ (pH 6.8). Cu foam was deposited at graphene sheet at 1 A cm^{-2} for 20 s. CVs start from OCP, forward scan potential limitation -0.7 V, back scan potential limitation +0.8 V. a) Representative time-series of XANES spectra of Cu foam at -0.2 V, the inset figure provides a zoomed-in view of the energy range around 8982 eV. b) The first 2 CV cycles of Cu foam. Normalized fluorescence at 8982 eV (red) and in-situ CV data and for c) cycle 1 and d) cycle 2. Normalized fluorescence at 9013 eV (red) and in-situ CV data and for e) cycle 1 and f) cycle 2. Arrows indicate the scan direction. For 9013 eV, the fluorescence intensity drops down to 0.93-0.94, indicating that the reduction process is more complete compared to the fast reduction at -0.7 V (with a fluorescence value of 0.95), see Supplementary Figure 18 and Supplementary Table 2, but likely also not fully complete (with a fluorescence value of 0.92 for the 10s-deposited/air-dried sample reduced at -0.2 V).

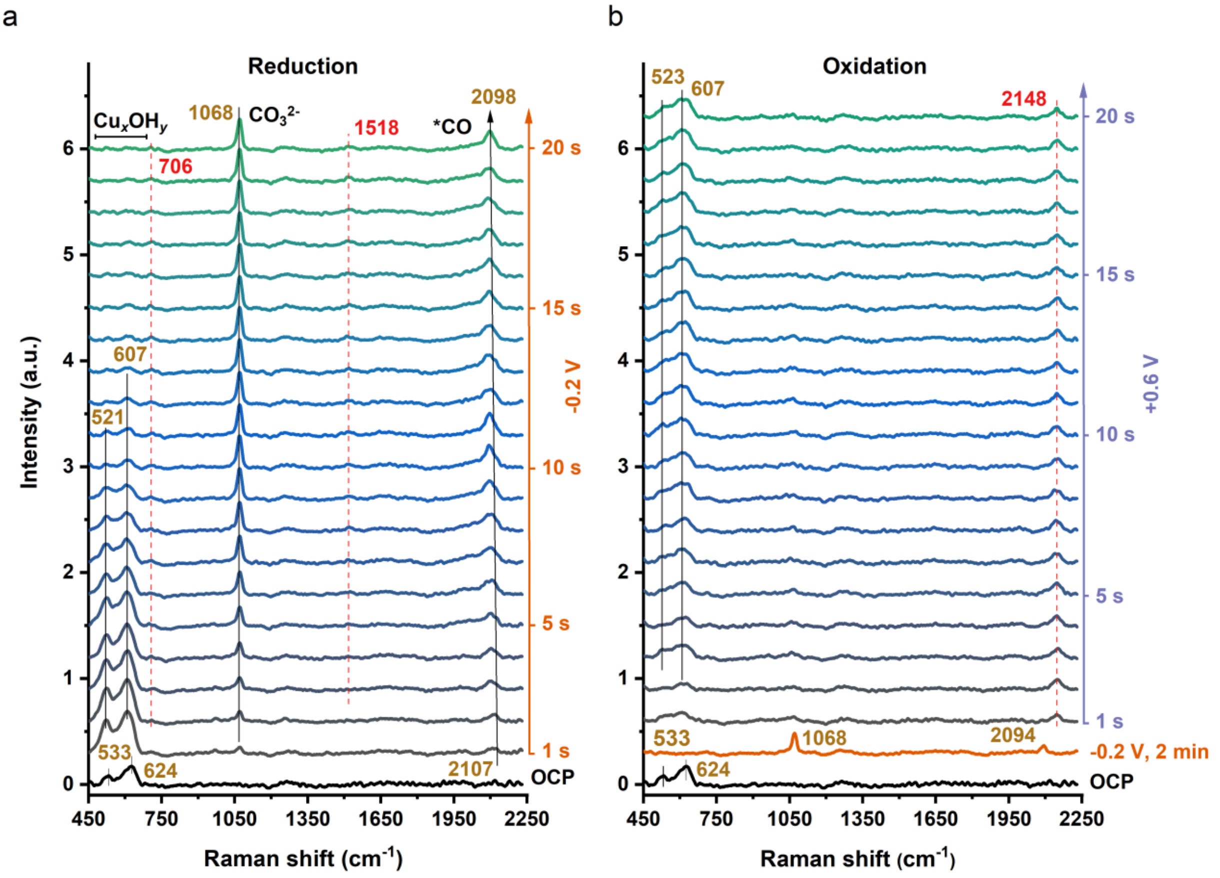

Supplementary Figure 22. Complete spectra of **Fig. 6**. Cu foam was deposited on Cu foil for 20 s at 1 A cm^{-2}. Operando SERS spectra collected at a) -0.2 V and b) +0.6 V in CO_2-saturated 0.1 M $KHCO_3$ (pH 6.8). The weak band at 706 cm^{-1} is assigned to in-plane bending of carbonate, the band at 1518 cm^{-1} is assigned to antisymmetric stretching of carbonate[2]. The origin of the 2148 cm^{-1} band is unclear.

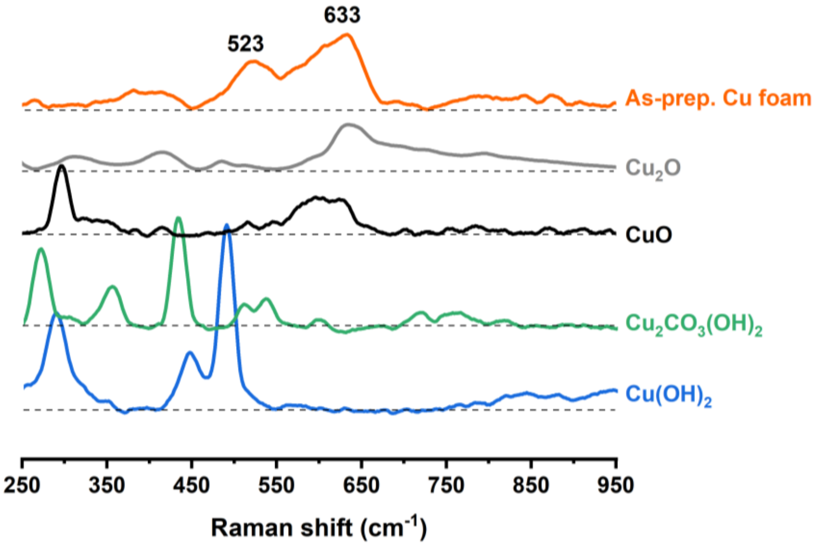

Supplementary Figure 23. Raman spectra of as-prepared Cu foam at OCP and spectra of Cu_2O, CuO, $Cu_2CO_3(OH)_2$ and $Cu(OH)_2$ powder samples shown for comparison (The black dash line represents the baseline). Cu foam was deposited on Cu foil at 1 A cm^{-2} for 20 s.

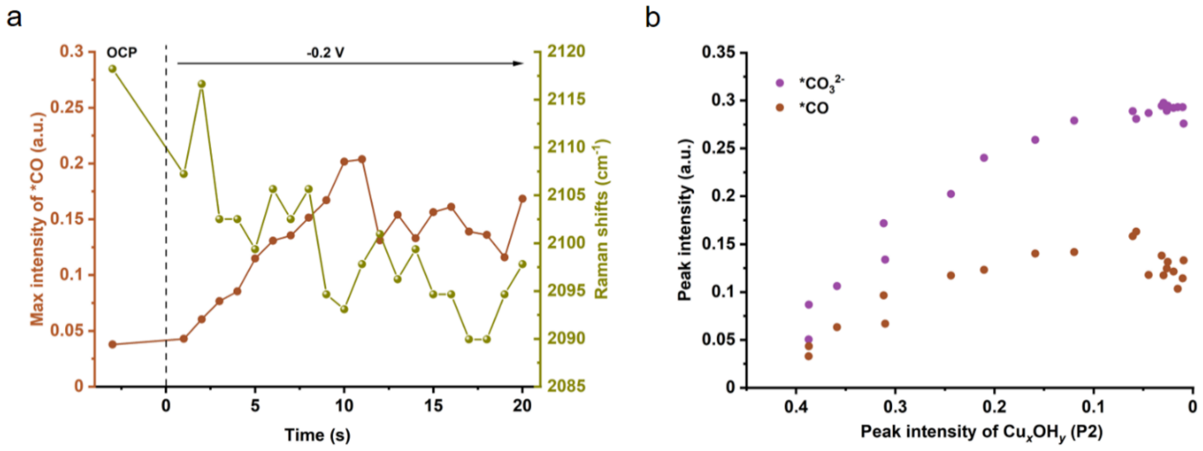

Supplementary Figure 24. Analysis of SERS data in Supplementary Figure 22. a) Time-dependence of the Raman shift and maximum intensity of the *CO vibration. b) Relations between Cu_xOH_y and adsorbed $*CO_3^{2-}$ and *CO band intensities. The Cu_xOH_y P2 peak was selected as the reference.

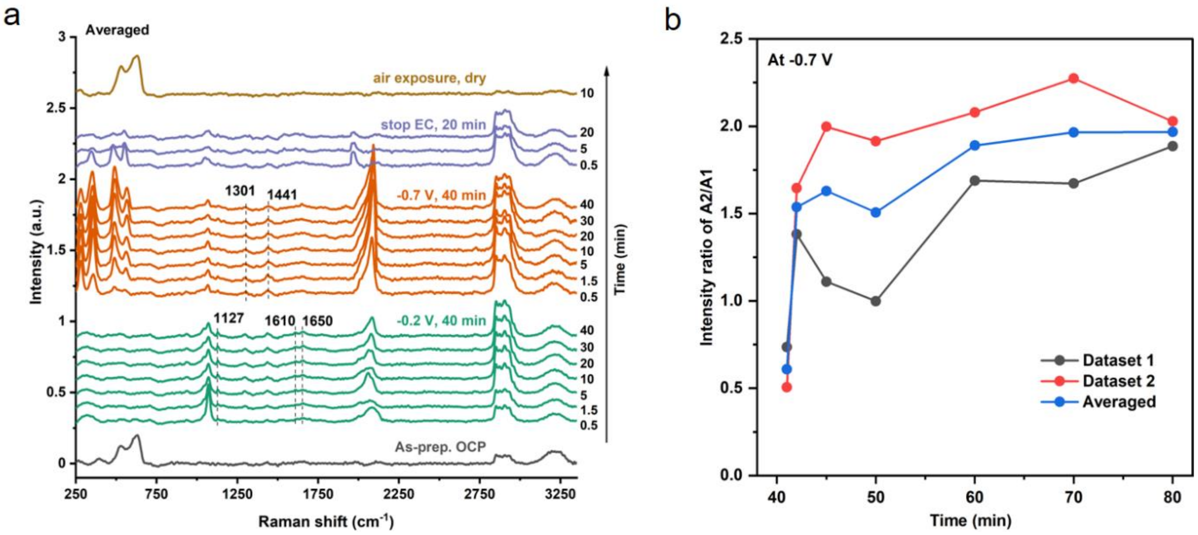

Supplementary Figure 25. a) Complete spectra of **Fig. 7a**. Spectra are averaged by two datasets in Supplementary Figure 26. Weak bands around 1127, 1301, and 1441 cm^{-1} are tentatively assigned to the carbonaceous species formed by electroreduction of irreversibly adsorbed CO_2 contaminations of the initial surface, as also observed in an earlier study[3]. The band at 1610 cm^{-1} could be assigned to the interfacial water vibration[4, 5]; while the band at 1650 cm^{-1} is likely a result of the C=O vibration[5, 6]. A broad band in the region of 2800-3000 cm^{-1} is assigned to the C-H vibration and a water peak is located at 3000-3300 cm^{-1} according to our previous study[7]. b) Time dependence of the intensity ratio of A2/A1 at -0.7 V. Data are taken from **Fig. 7b** and Supplementary Figure 26b and d.

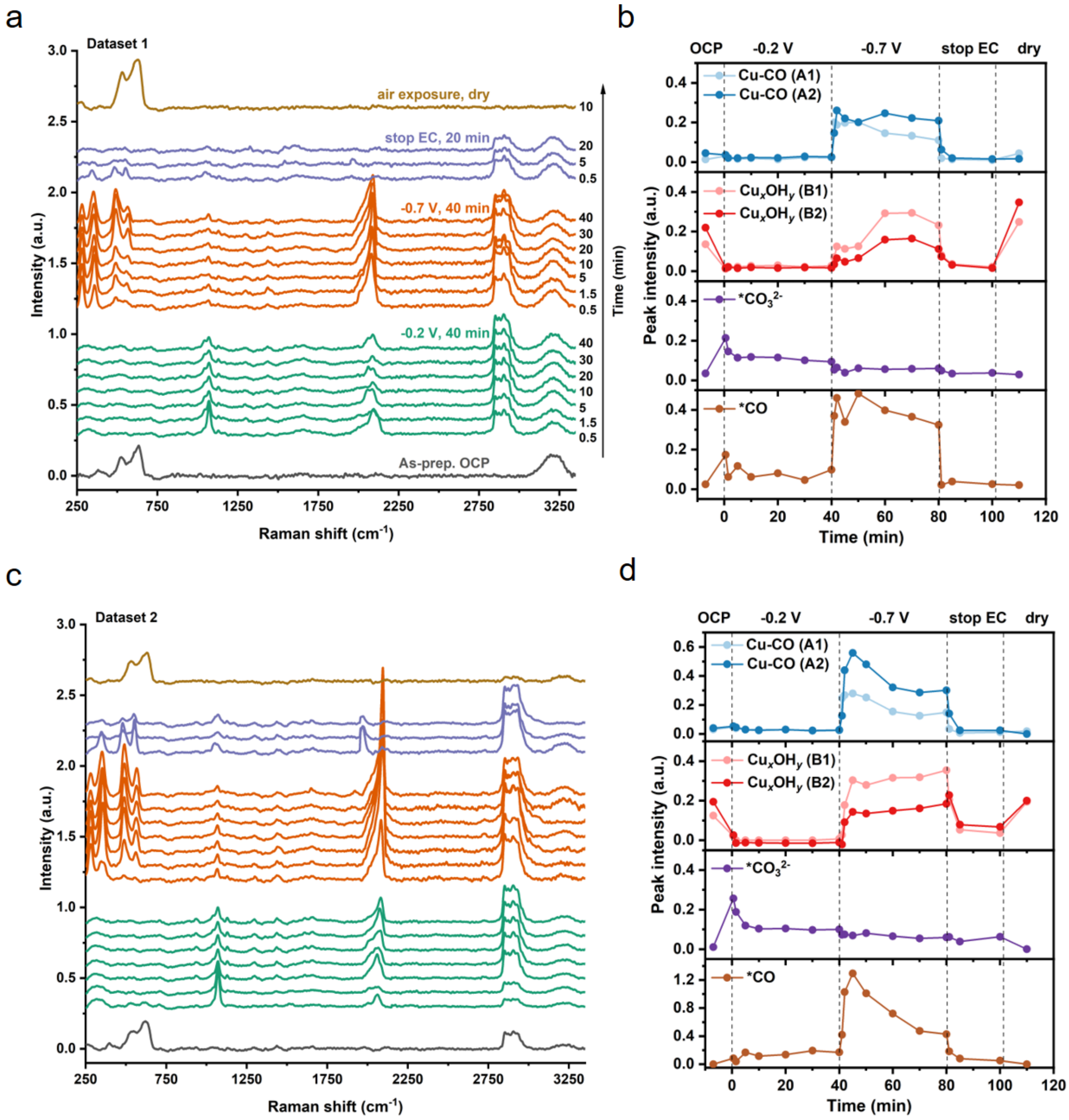

Supplementary Figure 26. Data were collected at OCP and during application of the indicated potentials in CO_2-saturated 0.1 M $KHCO_3$ (pH 6.8). Two datasets of Raman spectra in a) and c) and the respective time-traces of Cu-CO (A1, A2), Cu_xOH_y (B1, B2 or B1*, B2*), adsorbed $*CO_3^{2-}$ and *CO bands intensities in b) and d). a) and c) have the same color code. Grey: OCP; green: -0.2 V for 40 min; orange: -0.7 V for 40 min; purple: "stop EC" for 20 min (no potential applied, OCP condition); dark yellow: air-exposed Cu foam in the "dry" state.

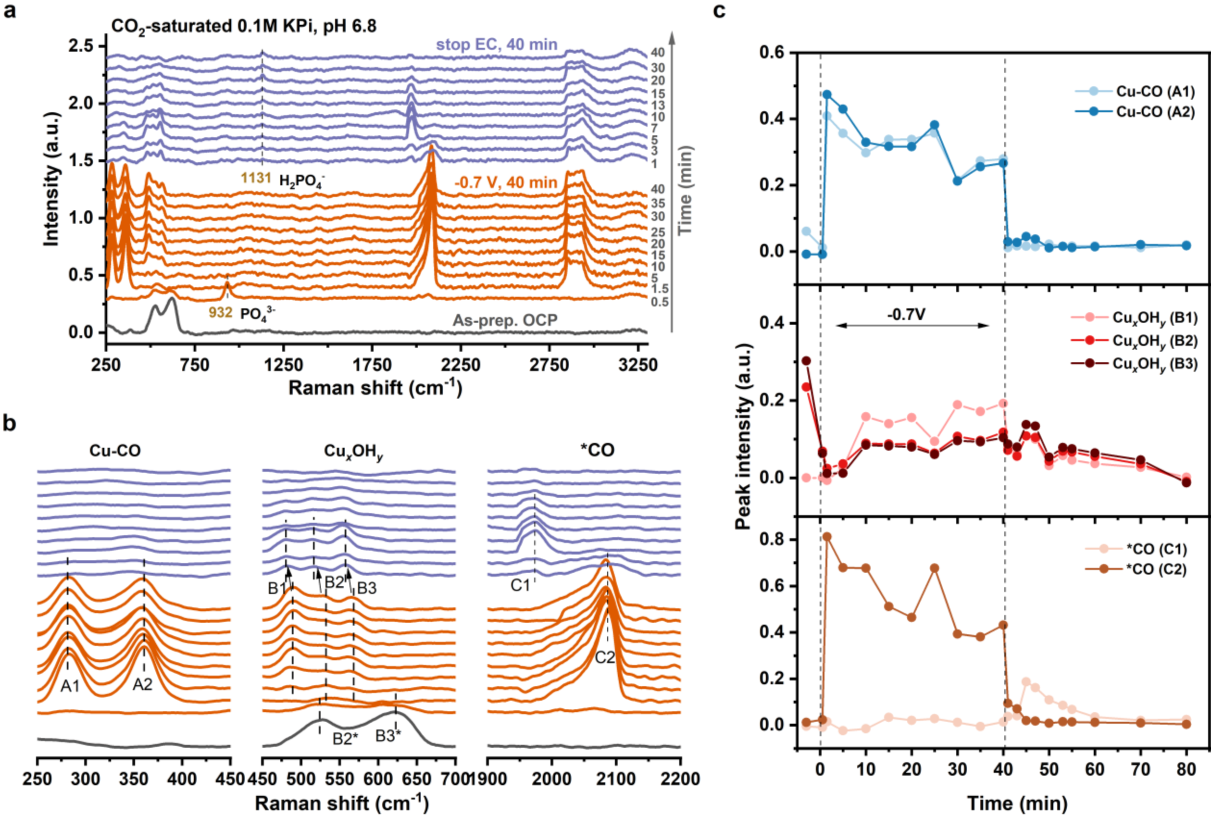

Supplementary Figure 27. Operando Raman spectra of Cu foam deposited on Cu foil. Co foam was deposited at 1 A cm^{-2} for 20 s in CO_2-saturated 0.1 M KPi solution (pH 6.8). a) Raman spectra of Cu foam at OCP (light black), -0.7 V for 40 min (orange) and "stop EC" for 40 min (without applied potential, purple line). b) Enlarged vibrational bands of Cu-CO, Cu_xOH_y and adsorbed *CO. The color code in b) is the same as in a). c) Time-courses of maximum peak intensity of Cu-CO (A1, A2), Cu_xOH_y (B1, B2, B3), and adsorbed *CO (C1, C2).

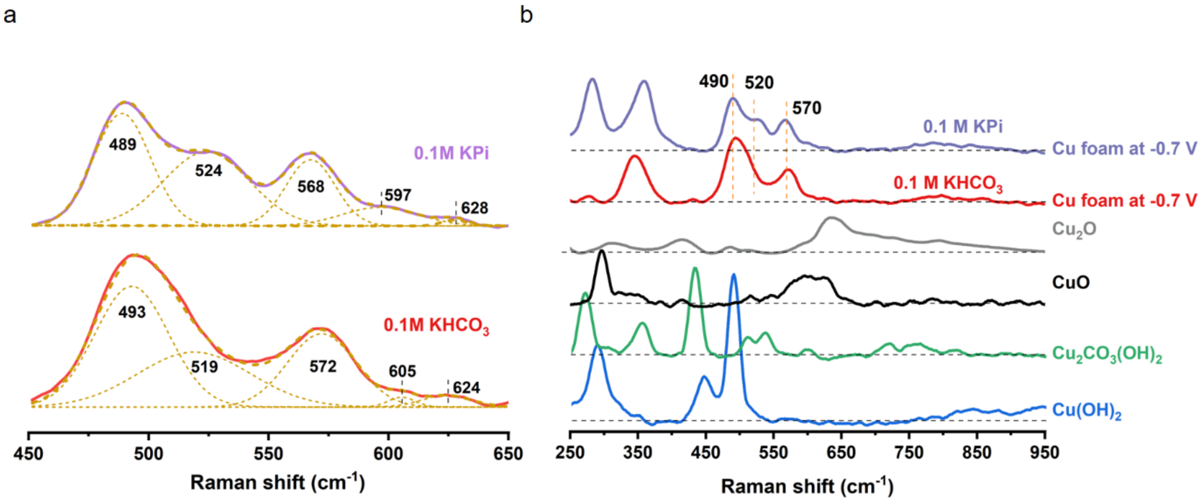

Supplementary Figure 28. Selected Raman spectra of Cu foam deposited on Cu foil at 1 A cm^{-2} for 20 s. a) Spectra of Cu foam in CO_2-saturated 0.1 M $KHCO_3$ (solid red line) and CO_2-saturated 0.1 M KPi solution (solid purple line) reacted at -0.7 V for 30 min (dashed lines show simulations with Gaussian functions). b) Spectra of Cu foam in CO_2-saturated 0.1 M $KHCO_3$ (red line) and CO_2-saturated 0.1 M KPi (purple line) electrolytes reacted at -0.7 V (potential was applied for 30 min prior to collection of

spectra); spectra of Cu_2O, CuO, $Cu_2CO_3(OH)_2$ and $Cu(OH)_2$ powder samples are shown for comparison. The black dashed line represents the baseline.

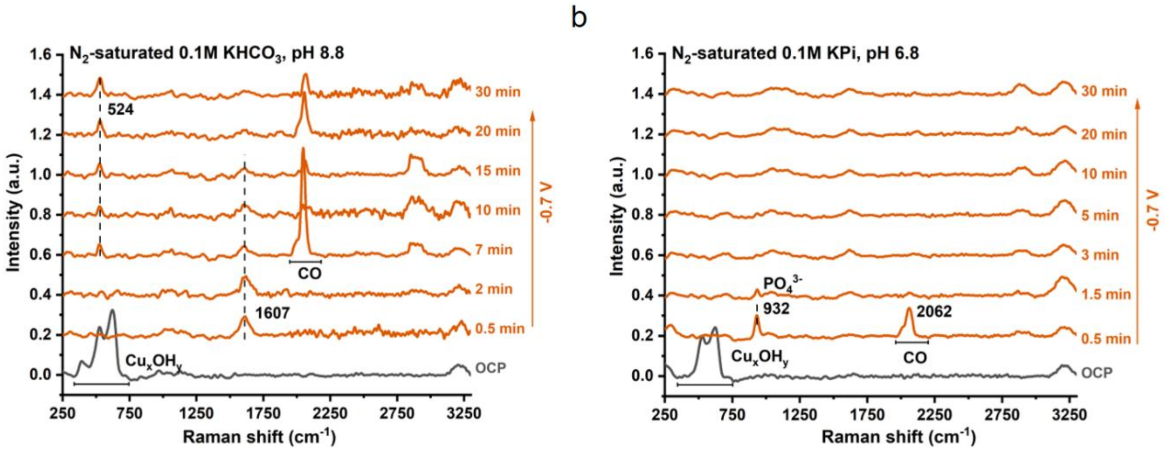

Supplementary Figure 29. Operando Raman spectra of Cu foam collected in a) N_2-saturated 0.1 M $KHCO_3$ (pH 8.8) and b) N_2-saturated 0.1 M KPi (pH 6.8). Cu foam was deposited on Cu foil at 1 A cm^{-2} for 20 s. a) The broad peak at 1607 cm^{-1} could be assigned to the interfacial water vibration[4], which is more pronounced in N_2-saturated $KHCO_3$ than in CO_2-saturated $KHCO_3$. b) The absence of the 1070 cm^{-1} band in CO_2-free solutions further confirms the assignment of the $^*CO_3$ vibration[2,7]. Only deprotonated PO_4^{3-} is present instead of $H_2PO_4^-$ and HPO_4^{2-}, suggesting pronounced alkalization at the electrode-electrolyte interface. The unexpected initial *CO peak may be derived from carbonate species in as-prepared Cu foam or trace amounts of CO_2 dissolved in the electrolyte.

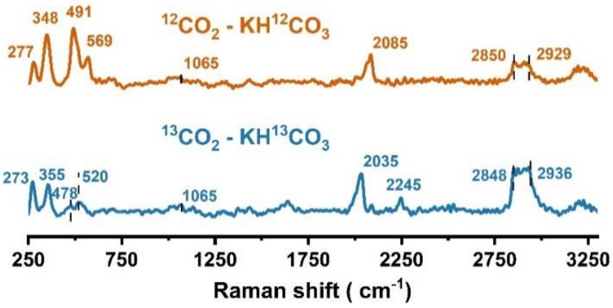

Supplementary Figure 30. Effect of isotope exchange on Raman spectra of Cu foam. Cu foam was deposited on Cu foil for 20 s with 1 A cm^{-2}. Spectra were recorded at a potential of -0.7 V in $^{12}CO_2$-saturated 0.1 M $KH^{12}CO_3$ (orange line) or in $^{13}CO_2$-saturated 0.1 M $KH^{13}CO_3$ (blue line). The adsorbed *CO band located at 2085 cm^{-1} is red-shifted to 2035 cm^{-1} in $KH^{13}CO_3$ and a new peak at 2245 cm^{-1} detected in the labeled electrolyte is assigned to solution-phase $^{13}CO_2$[4, 8]. The restricted rotation band of adsorbed CO at 277 cm^{-1} shifts to 273 cm^{-1}, and the Cu-CO stretching band at 348 cm^{-1} displays a blue shift to 355 cm^{-1} in $KH^{13}CO_3$ electrolyte. These observations are in good agreement with previous studies[8, 9]. The CO_3^{2-} adsorption peak at 1065 cm^{-1} is hardly affected by $^{13}C/^{12}C$ isotope exchange, which is due to the centrosymmetric movements of the oxygen atoms with respect to the central carbon atom. The broad peak at 2840-2940 cm^{-1}, assigned to C-H stretching, remained unchanged due to the overlap of various $-CH_x$ bands.

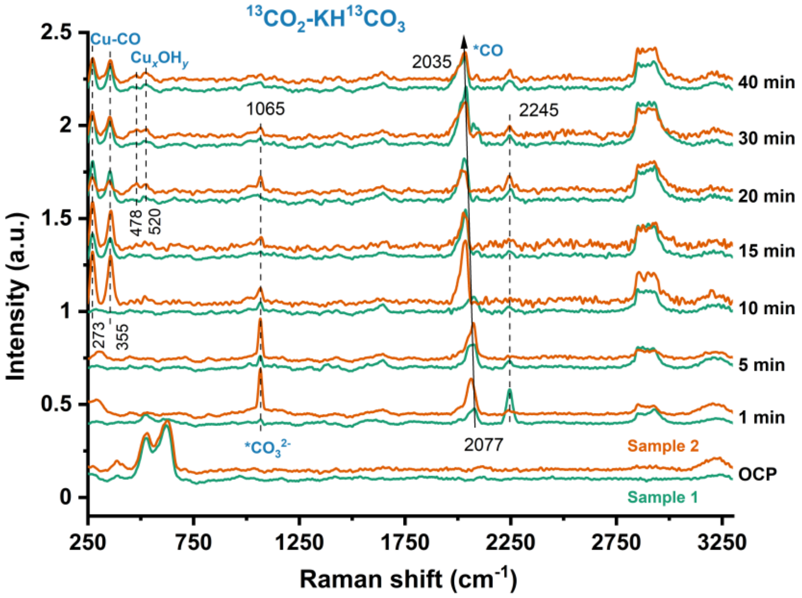

Supplementary Figure 31. Operando Raman spectra of Cu foam collected in $^{13}CO_2$-saturated 0.1 M KH$^{13}CO_3$. Cu foam was deposited on Cu foil at 1 A cm^{-2} for 20 s. Comparison of spectra of independently prepared samples 1 (green line) and 2 (orange line) shows that the band shifts induced by ^{12}C /^{13}C exchange can be qualitatively reproduced.

Supplementary Table 3. Assignments of vibrational bands of adsorbates on copper catalysts during CO_2RR

metal	band position (cm^{-1}) and vibration mode assignment		
Cu[10]	502 (Cu-C)	525 (Cu-OH)	
Cu[11]		530 (Cu-OH)	
Cu[12]		529 (*CH_2CHO/Cu-C and Cu-O)	
Cu[13]		530 (*OH)	
Cu[14]		480 and 526 (C-containing species)	
Cu[15]	460 (Cu-C)		
Cu[16]	493 (Cu(CO)$_2$)		
Cu (this work)	493 (Cu-C)	519 (Cu-OH)	572 (C-containing species

5 Supplementary Notes

5.1 Supplementary Note 1

Interpretation of Raman bands in the region of 450-600 cm^{-1} in light of recent literature and experimental results

In our study, additional Raman bands in the range of 450-600 cm^{-1} appeared at negative potential of -0.7 V during CO_2RR. Although previous assignments[5, 10, 13-18] associated these bands with CuO_xH_y species, the rationale behind this assignment has not been fully justified. Subsequently, we consider three possible pathways for the formation of the CuO_xH_y species: (1) the species originate from the original Cu oxides present in the as-prepared sample; (2) they arise from a reaction between reduced Cu and H_2O; and (3) they arise from a reaction between reduced Cu and CO_2-saturated electrolyte.

As shown in **Fig. 7**, the original oxide species in Cu foam were firstly reduced at -0.2 V, followed by the formation of CuO_xH_y at -0.7 V. The formation of CuO_xH_y is both potential and time-dependent. Therefore, it can be ruled out that the CuO_xH_y species originated from the original oxides in the as-prepared sample (pathway 1). This conclusion is further supported by Chen *et al.*[18], who conducted a CO_2RR experiment using ^{18}O-labeled OD-Cu catalysts. The CuO_xH_y bands in the ^{18}O system exhibited negligible shifts compared to those of non-labeled OD-Cu, supporting the finding that the formed CuO_xH_y species did not come from the original OD-Cu catalyst.

Regarding the second pathway, Chen *et al.*[18] performed the CO_2RR on OD-Cu catalysts in the electrolyte containing only $H_2{}^{18}O$. The bands of interest did not exhibit any shifts, ruling out the possibility of CuO_xH_y formation from a reaction between Cu and $H_2{}^{18}O$. This conclusion is further supported by the spectra measured in N_2-saturated KPi (phosphate buffer) in our study (Supplementary Figure S29b). No related CuO_xH_y bands were formed in the carbon-free electrolyte, suggesting that the carbon-containing nature of the bands or their formation depends on reduction of CO_2.

From the Raman spectra collected in CO_2-saturated KPi (pH 6.8), and CO_2-saturated $KHCO_3$ electrolyte (pH 6.8), three main peaks in the 450-600 cm^{-1} region were identified by Gaussian fitting. The second peak around 520 cm^{-1} showed negligible shifts during $^{12}C/^{13}C$ isotope exchange ($^{13}CO_2$-saturated $KH^{13}CO_3$) and was also present in N_2-saturated $KHCO_3$ electrolyte (pH 8.8). These results suggest that the band at 520 cm^{-1} is independent of carbon and likely promoted at more alkaline pH. This finding aligns with the 520 cm^{-1} band assigned to a Cu-OH vibration, as suggested in previous studies[10, 11, 13]. However, the first peak at 493 cm^{-1} and the third peak at 572 cm^{-1} are both affected by the $^{12}C/^{13}C$ isotope exchange and are absent in the absence of CO_2 saturation. Therefore, we suggest that these peaks are C-associated vibrations, as further discussed below.

5.2 Supplementary Note 2

The nature of C-containing species

In **Fig.8b**, the first peak at 493 cm^{-1} aligns with the Cu-C vibration[10, 16]. The adsorption of *COOH on Ag[17] exhibited the Ag-C vibration at 408 cm^{-1}, accompanied by the C=O vibration at 1660 cm^{-1}. The similar Raman band at around 1650 cm^{-1} is also observed in Supplementary Figure 25a. Consequently, it is plausible to assign the 493 cm^{-1} band to the Cu-C vibration arising from the adsorption of *COOH. Finally, we attempted to assign the third peak at 572 cm^{-1}. The DFT-calculated vibrational frequency[19] of CO_2/Cu(111) was reported as 580 cm^{-1}; the adsorption of $*OCO^-$ on Ag[5] exhibited a vibrational frequency of 530 cm^{-1}. The protonated $*OCO^-$ (*OCHO) is widely considered as a key intermediate for the formation of formate[6, 20, 21]. Since formate is also one of the CO_2RR products as shown in Supplementary Figure 2, it is highly likely that $*OCO^-$ is present in the local alkaline environment. The aforementioned facts allowed us to associate the 572 cm^{-1} band with a "O-C-O" vibration. However,

various C-containing intermediates are formed during the CO_2RR. Therefore, the definitive assignment of these two bands requires further extensive experimental and theoretical investigations.

5.3 Supplementary Note 3

The adsorption of *OH at negative potentials

As suggested in Ref[22-24], as well as corroborated by our study, the presence of subsurface oxygen species in the OD-Cu catalysts result in the formation of Cu^{+}/Cu^{2+}. This formation enhances the adsorption of *CO and *OH through the electrostatic interactions.

In our earlier investigations, we observed a pronounced alkalization[7] (pH>10) at negative potentials during CO_2RR, as well as a large potential gradient[9] in the first layer of the adsorbed material (Helmholtz layer), causing the potential of the bulk electrolyte to be around 1.3 V more positive than the applied potential. Consequently, even at a potential of -0.7 V, the electrolyte potential can become positive enough, particularly in conjunction with local alkalization, thereby promoting the formation of oxidized species such as Cu hydroxides.

6 Supplementary References

1. Klingan, K. et al. Reactivity Determinants in Electrodeposited Cu Foams for Electrochemical CO_2 Reduction. *ChemSusChem* **11**, 3449-3459 (2018).
2. Jiang, S., D'Amario, L. & Dau, H. Copper Carbonate Hydroxide as Precursor of Interfacial CO in CO_2 Electroreduction. *ChemSusChem* **15**, e202102506 (2022).
3. Chernyshova, I.V., Somasundaran, P. & Ponnurangam, S. On the origin of the elusive first intermediate of CO_2 electroreduction. *Proc. Natl. Acad. Sci. U.S.A.* **115**, E9261-E9270 (2018).
4. Nikolic, B.Z. et al. Electroreduction of carbon dioxide on platinum single crystal electrodes: electrochemical and in situ FTIR studies. *J. Electroanal. Chem. Interf. Electrochem.* **295**, 415-423 (1990).
5. Shan, W. et al. In Situ Surface-Enhanced Raman Spectroscopic Evidence on the Origin of Selectivity in CO_2 Electrocatalytic Reduction. *ACS Nano* **14**, 11363-11372 (2020).
6. Bohra, D. et al. Lateral adsorbate interactions inhibit HCOO− while promoting CO selectivity for CO_2 electrocatalysis on silver. *Angew. Chem., Int. Ed.* **131**, 1359-1363 (2019).
7. Jiang, S., Klingan, K., Pasquini, C. & Dau, H. New aspects of operando Raman spectroscopy applied to electrochemical CO_2 reduction on Cu foams. *J. Chem. Phys.* **150**, 041718 (2018).
8. Zhu, S., Li, T., Cai, W.-B. & Shao, M. CO_2 Electrochemical Reduction As Probed through Infrared Spectroscopy. *ACS Energy Lett.* **4**, 682-689 (2019).
9. Zhu, S., Jiang, B., Cai, W.-B. & Shao, M. Direct Observation on Reaction Intermediates and the Role of Bicarbonate Anions in CO_2 Electrochemical Reduction Reaction on Cu Surfaces. *J. Am. Chem. Soc.* **139**, 15664-15667 (2017).
10. Moradzaman, M. & Mul, G. In Situ Raman Study of Potential-Dependent Surface Adsorbed Carbonate, CO, OH, and C Species on Cu Electrodes During Electrochemical Reduction of CO_2. *ChemElectroChem* **8**, 1478-1485 (2021).
11. Nguyen, T.N. et al. Catalyst Regeneration via Chemical Oxidation Enables Long-Term Electrochemical Carbon Dioxide Reduction. *J. Am. Chem. Soc.* **144**, 13254-13265 (2022).
12. Zhao, Y. et al. Elucidating electrochemical CO_2 reduction reaction processes on Cu(hkl) single-crystal surfaces by in situ Raman spectroscopy. *Energy Environ. Sci.* **15**, 3968-3977 (2022).
13. Jeon, H.S. et al. Selectivity Control of Cu Nanocrystals in a Gas-Fed Flow Cell through CO_2 Pulsed Electroreduction. *J. Am. Chem. Soc.* **143**, 7578-7587 (2021).
14. Lei, Q. et al. Structural evolution and strain generation of derived-Cu catalysts during CO_2 electroreduction. *Nat. Commun.* **13**, 4857 (2022).
15. de Ruiter, J. et al. Probing the Dynamics of Low-Overpotential CO_2-to-CO Activation on Copper Electrodes with Time-Resolved Raman Spectroscopy. *J. Am. Chem. Soc.* **144**, 15047-15058 (2022).
16. Alikhani, M.E. & Manceron, L. The copper carbonyl complexes revisited: Why are the infrared spectra and structures of copper mono and dicarbonyl so different? *J. Mol. Spectrosc.* **310**, 32-38 (2015).
17. Shan, W., Liu, R., Zhao, H. & Liu, J. Bicarbonate Rebalances the *COOH/*OCO^- Dual Pathways in CO_2 Electrocatalytic Reduction: In Situ Surface-Enhanced Raman Spectroscopic Evidence. *J. Phys. Chem. Lett.* **13**, 7296-7305 (2022).
18. Chen, C. et al. The in situ study of surface species and structures of oxide-derived copper catalysts for electrochemical CO_2 reduction. *Chem. Sci.* **12**, 5938-5943 (2021).
19. Chernyshova, I.V., Somasundaran, P. & Ponnurangam, S. On the origin of the elusive first intermediate of CO_2 electroreduction. *Proc. Natl. Acad. Sci. U.S.A.* **115**, E9261-E9270 (2018).
20. Liu, F. et al. Inhibiting Sulfur Dissolution and Enhancing Activity of SnS for CO_2 Electroreduction via Electronic State Modulation. *ACS Catal.* **12**, 13533-13541 (2022).
21. Ma, W. et al. Promoting electrocatalytic CO_2 reduction to formate via sulfur-boosting water activation on indium surfaces. *Nat. Commun.* **10**, 892 (2019).

22. Favaro, M. et al. Subsurface oxide plays a critical role in CO_2 activation by Cu(111) surfaces to form chemisorbed CO_2, the first step in reduction of CO_2. *Proc. Natl. Acad. Sci. U.S.A.* **114**, 6706-6711 (2017).
23. Wang, H.-Y. et al. Direct Evidence of Subsurface Oxygen Formation in Oxide-Derived Cu by X-ray Photoelectron Spectroscopy. *Angew. Chem., Int. Ed.* **61**, e202111021 (2022).
24. Fields, M., Hong, X., Nørskov, J.K. & Chan, K. Role of Subsurface Oxygen on Cu Surfaces for CO_2 Electrochemical Reduction. *J. Phys. Chem. C* **122**, 16209-16215 (2018).
